# Flame Synthesized Co–CeO_2_ Catalysts for CO_2_ Methanation

**DOI:** 10.1021/acscatal.5c02380

**Published:** 2025-06-13

**Authors:** Angelina Evtushkova, Jason M.J.J. Heinrichs, Alexander Parastaev, Nikolay Kosinov, Emiel J. M. Hensen

**Affiliations:** Laboratory of Inorganic Materials and Catalysis, Department of Chemical Engineering and Chemistry, Eindhoven University of Technology, 5600 MB Eindhoven, The Netherlands

**Keywords:** CO_2_ methanation, CeO_2_, Co, flame spray pyrolysis, Co−O−Ce interface, oxygen vacancies

## Abstract

Achieving selective conversion of CO_2_ to CO, CH_4_, or CH_3_OH remains a key challenge in catalyst design for CO_2_ hydrogenation. Site-specific activity at the metal–support interface plays a crucial role, motivating efforts to optimize metal particles and their interactions with supports. In this study, we synthesized Co-CeO_2_ catalysts with varying Co contents via flame spray pyrolysis (FSP) to investigate how the location and structure of Co influence activity. All samples contain ∼8 nm CeO_2_ nanoparticles with a high surface area and approximately 3.8 mol % Co^2+^ ions strongly interacting with CeO_2_. Catalysts with ≥5 mol % Co feature segregated CoO and Co_3_O_4_ particles, which are partially reduced to metallic Co at 300 °C. The highest Co-weight-normalized activity at 200 °C (3.9 ± 0.2 mmol CO_2_/mol Co/s, CH_4_ selectivity 85%) was observed in 10 mol % Co-CeO_2_, with ∼50% Co reduction and 4–5 nm Co nanoparticles. The 2.5 mol % Co sample exhibited only 10% reduction, forming small Co clusters and creating Co^2+^–O–Ce sites that mainly favor CO formation (79% selectivity). Low Co content facilitates CO_2_ hydrogenation to CO and minor CH_3_OH formation, likely on oxygen vacancies, assisted by H_2_ dissociation on very small metallic Co clusters. Larger Co nanoparticles predominantly produce CH_4_, with minor CO and no CH_3_OH. These results demonstrate that FSP enables tuning of catalyst structures for selective CO_2_ hydrogenation, leveraging the synergy between small metallic Co particles and Co^2+^–O–Ce sites.

## Graphical abstract

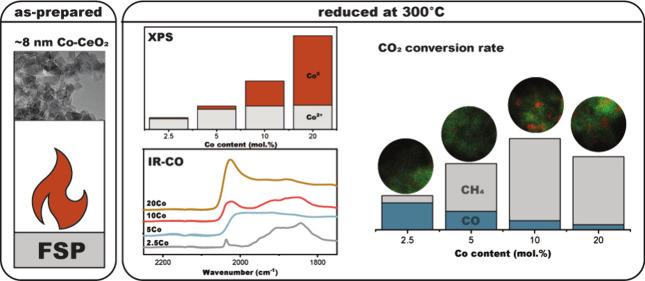

## Introduction

Carbon dioxide (CO_2_) is a greenhouse gas that is anthropogenically released into the atmosphere, contributing to climate change and ocean acidification. Mitigation of the net flow of CO_2_ into the atmosphere can be achieved by (i) reducing CO_2_ emissions, (ii) CO_2_ capture and storage, and (iii) utilization of (captured) CO_2_.^1–3^ CO_2_ hydrogenation with H_2_ obtained from renewable energy sources into fuels and chemicals is a promising approach to close carbon cycles. Converting CO_2_ to methane (CH_4_) is a viable technology for reusing CO_2_ by temporarily storing hydrogen (H_2_) in a widely used energy carrier.^4,5^ CH_4_ can be used directly in existing natural gas infrastructure in many countries.^6^ The chemical transformation of CO_2_ can be done via electrocatalytic,^7^ photocatalytic^8^ and thermocatalytic reduction of CO_2_.^9^ The latter approach is the most developed for sustainable CH_4_ production from CO_2_ and renewable energy. Catalyst design approaches can enhance the performance of heterogeneous catalysts for converting CO_2_ into CH_4_ through hydrogenation.

Catalysts based on first-row transition metals have garnered particular interest owing to their abundance and lower cost compared to noble metals. Copper (Cu) is predominantly used for the heterogeneous hydrogenation of CO_2_ to methanol (CH_3_OH).^10,11^ Nickel (Ni) catalysts are most extensively studied for CO_2_ methanation due to their high activity.^12–14^ Although iron (Fe) catalysts are preferentially employed in Fischer–Tropsch synthesis (FTS) from CO_2_/H_2_ mixtures,^15^ cobalt (Co) also presents significant interest for converting CO_2_ to higher hydrocarbons^16^ or higher alcohols,^17^ attributable to its high activity in FTS from CO/H_2_.^18–21^ In practice, the conversion of CO_2_ to hydrocarbons using Co catalysts often proceeds via the reverse water–gas shift (rWGS) reaction, followed by CO-FTS. Given Co’s superior ability for C–O bond scission relative to Ni, exploring Co for CO_2_ methanation merits consideration. Although typically considered less active, a recent review noted that Co catalysts outperform Ni ones at lower reaction temperatures.^22^

The support plays a critical role in the formation of active sites in heterogeneous catalysts.^23^ Liang et al. showed that a difference in metal–support interactions led to a higher activity of Co/Al_2_O_3_ in CO_2_ methanation than Ni/Al_2_O_3_.^24^ Interactions between Co and reducible oxide supports (e.g., CeO_2_ and TiO_2_) are particularly interesting. Fu et al. demonstrated that the combination of the CoO-CeO_2_ and CoO-Co interfaces is very active in the water–gas shift (WGS) reaction.^25^ Wang et al. showed that silica-supported metallic Co nanoparticles are active in CO_2_ hydrogenation to CO and CH_4_, whereas Co–O–SiO_n_ structures close to metallic Co can catalyze CO_2_ hydrogenation to CH_3_OH in catalysts with strong Co-silica interactions.^26^ Another study of CO_2_ hydrogenation concluded that CoO on TiO_2_ results in higher CO_2_ conversion and C_2+_ selectivity caused by H-assisted CO_2_ dissociation compared to conventional Co metal nanoparticles on TiO_2_.^16^ Parastaev et al. showed that small Co clusters dispersed on the surface of CoO nanoparticles, which are stabilized by a CeO_2_–ZrO_2_ support, are much more active in CO_2_ methanation than metallic Co particles.^27^ The use of reducible supports is a promising approach to promote CO_2_ conversion by metal phases due to their unique redox properties and enhanced metal–support interactions.^28^ The design of Co-CeO_2_ catalysts with tailored structures, such as interfacial sites containing oxygen vacancies and Co nanoparticles, has emerged as a promising strategy for preparing highly active catalysts for CO_2_ hydrogenation. However, despite significant progress, the precise engineering and control of the Co-CeO_2_ interface, which is critical for optimizing metal–support interactions, remains a considerable challenge.

Catalyst preparation usually involves wet methods, such as wet impregnation, to load Co on a support.^29^ An inherent limitation of this method is the relatively low Co loading that can be achieved.^30^ Other conventional wet methods, such as coprecipitation, lack control over the particle size.^30^ Although metal doping in CeO_2_ can be achieved in this way, it is often inhomogeneous because of the different pH values at which Ce and Co ions precipitate. This method also requires large amounts of solvent to remove the precipitation agent. Flame spray pyrolysis (FSP) is a versatile method to prepare well-defined nanosized catalysts, offering flexibility in terms of composition, precise particle size control, and scalability.^31–33^ FSP involves injecting a solution of the metals of interest into a methane-oxygen flame (Figure S1), the high temperatures limiting sintering. As the solvent and organic ligands of the metal precursors are completely burned in the flame, the resulting catalysts can be used directly without additional drying or calcination steps. Solubilizing different metal salts in suitable organic solvents contributes to a homogeneous mixing of the metal ions in the composite metal oxides.^33^ Typically, the compositional range can be very broad in such catalysts.^34,35^

This study examines a series of Co–CeO_2_ catalysts prepared by one-step FSP to investigate the effect of Co–CeO_2_ structure on CO_2_ methanation. The catalytic activity determined in the 200–300 °C temperature range and atmospheric pressure showed that catalysts with a low Co content mainly produced CO at a low CO_2_ conversion, while samples with a high Co content presented CH_4_ formation at much higher CO_2_ conversion. The as-prepared and reduced catalysts were extensively characterized by N_2_ physisorption, transmission electron microscopy (TEM), X-ray diffraction (XRD), temperature-programmed reduction (H_2_-TPR), near-ambient pressure X-ray photoelectron spectroscopy (NAP-XPS), and X-ray absorption spectroscopy (XAS). Structural changes of the catalysts during reductive pretreatment were monitored by in situ synchrotron X-ray diffraction (XRD) in combination with real-space-based pair distribution function (PDF) analysis. Partial reduction of the CeO_2_ surface was observed in all Co-containing CeO_2_ samples, indicating a higher abundance of surface oxygen vacancies than the bare FSP-prepared CeO_2_ support. The high activity and CH_4_ selectivity of catalysts with a Co content >5 mol % is due to the formation of Co metal nanoparticles during reduction. On the other hand, samples with a lower Co content are hardly reduced, with only a very small amount of metallic Co in the form of clusters. This work is also available as part of a PhD thesis.^36^

## Methods

### Materials

Co (II) acetylacetonate (Co (C_5_H_7_O_2_)_3,_ 97%, Sigma-Aldrich), glacial acetic acid (99% Sigma-Aldrich) and 2-ethylhexanoic acid (99% Fisher Scientific), cerium­(III) acetate hydrate (Ce (CH_3_CO_2_)_3_·1H_2_O, 98%, TCI Europe NV) were used as received without further purification.

### Catalyst Preparation

#### Flame Spray Pyrolysis

CeO_2_ and Co-CeO_2_ samples were prepared by flame spray pyrolysis (FSP) in a Tethis NPS10 apparatus. Appropriate amounts of Co­(C_5_H_7_O_2_)_3_ and Ce­(CH_3_CO_2_)_3_·H_2_O were dissolved in an equivolumetric solvent mixture of acetic acid and 2-ethylhexanoic acid. The Co and Ce concentrations were 0.15 M. This solution was stirred at 80 °C for approximately 1 h until the metal precursors were completely dissolved. The precursor solution was fed using a syringe pump (injection rate of 5 mL/min) through a nozzle to obtain a fine spray in the center of the flame. The pressure drop at the capillary tip was kept at 2.5 bar by adjusting the orifice gap area at the nozzle. The flame was maintained by a feed of 1.5 L/min methane and 3.0 L/min oxygen. Solid samples were collected on a glass microfiber filter (Whatman) using a membrane vacuum pump. The as-prepared CeO_2_ and Co–CeO_2_ catalysts are denoted as CeFSP and xCoFSP, where x stands for the molar Co content (mol %) with respect to the support (Co/(Co+Ce)).

#### Impregnation

A reference catalyst with an intended Ni loading of 10 mol % was prepared by wet impregnation. For this purpose, the desired amount of Ni­(NO_3_)_2_ was dissolved in 60 mL of an aqueous ammonia solution (28 wt %). Approximately 3 g of commercially available CeO_2_ was added to the solution, followed by stirring the suspension for 2 h. Then, water was removed by evaporation. The catalyst was dried in air at 110 °C overnight and calcined at 400 °C for 4 h. These samples were denoted as 10Ni/CeO_2_.

Another reference catalyst with an intended Ni loading of 10 mol % was prepared by incipient wetness impregnation using citric acid to control the dispersion of the Ni nanoparticles (citric acid/Ni ratio of 0.13).^37^ The SiO_2_ support was dried in air at 110 °C overnight before impregnation. The desired amounts of Ni­(NO_3_)_2_ and citric acid were dissolved in deionized water. The resulting solution was used for impregnation. The catalyst was dried in air overnight at 110 °C and then calcined at 400 °C for 4 h. This sample is denoted as 10Ni/SiO_2_.

### Catalyst Characterization

#### Inductively Coupled Plasma Optical Emission Spectroscopy (ICP-OES)

The elemental composition of the as-prepared catalysts was determined by ICP-OES analysis (Spectro CIROS CCD Spectrometer). Prior to the analytical measurements, the catalysts were dissolved in 5 mL of concentrated sulfuric acid (H_2_SO_4_) at 200 °C under stirring for at least 30 min, followed by dilution in water.

#### N_2_ Physisorption

The textural properties of the as-prepared catalysts were determined by N_2_ physisorption at a temperature of – 196 °C using a Micrometrics TriStar II 3020 instrument. Prior to the physisorption measurements, the samples were heated to 160 °C in a N_2_ flow for 4 h. The specific surface area (SSA) was determined using the Brunauer–Emmett–Teller (BET) method.

#### X-ray Diffraction (XRD)

X-ray powder diffraction patterns were collected at the ID31 beamline of the European Synchrotron Radiation Facility (ESRF, Grenoble, France). The measurements were performed with an incident X-ray energy of 68 keV using a 0.5 mm × 0.5 mm (H x V) X-ray beam in transmission mode using a DECTRIS Pilatus 3X CdTe 2 M detector. These measurements were done at room temperature. The samples were sealed in Kapton tubes (Goodfellow, 3 mm o.d., 0.03 mm wall thickness). Beeswax (Alfa) was used to seal the two ends of the tube. Detector broadening was calibrated using a CeO_2_ reference obtained from NIST. The diffraction patterns, specifically the presence of CeO_2_ and Co_3_O_4_ phases, were analyzed by Rietveld refinement using the GSAS software.^38^

In situ synchrotron X-ray diffraction patterns were collected at the ID15A beamline of the ESRF. The measurements were carried out in transmission mode using an incident X-ray energy of 100 keV. A Pilatus3X CdTe 2 M detector was used to collect the scattered signal. Approximately 20 mg of sieved catalyst (125–250 mm) was loaded into a quartz capillary (2 mm outer diameter, wall thickness 0.1 mm) and retained between two layers of glass wool. The capillary was sealed with PTFE ferrules in a home-built Clausen-type flow cell. The sample was heated using a gas blower (Cyberstar). The temperature was measured by a thin (0.25 mm) K-type thermocouple placed inside the catalyst bed. Typically, the temperature was raised from 50 to 300 °C at a rate of 8.5 °C/min in a flow of 50 mL/min of a mixture of 20 vol % H_2_ in Ar, followed by an isothermal dwell of 0.5 h at 300 °C. Then, the system was cooled to 250 °C in the same mixture. After reaching this temperature, the reduction mixture was replaced with a reaction mixture of 5 vol % CO_2_ and 20 vol % H_2_, balanced by Ar, and fed at a 50 mL/min flow rate for 0.5 h. The detector distance, energy, and tilt were calibrated using a standard CeO_2_ powder obtained from NIST. The CeO_2_ phase in these XRD data was analyzed by Rietveld refinement using the GSAS software. Pair distribution function (PDF) data up to q = 28 Å^–1^ were reduced from the XRD data using the pdfgetX3 software [36]. Real-space refinements were carried out using the PDFgui software.^39^ To this end, the PDF is described using the G­(r) formalism, which reflects the probability of finding a pair of atoms separated by a distance r with an integrated intensity dependent on the pair multiplicity and the scattering factors of the elements involved. G­(r) was experimentally determined by the Fourier transform of the total scattering function F­(Q), corresponding to the coherent scattering coming from the sample (Bragg peaks and diffuse scattering) after normalization.

#### Ultraviolet–Visible Diffuse Reflectance Spectroscopy (UV–vis DRS)

UV–vis DRS spectra were collected at room temperature using a Shimadzu UV-2401PC spectrometer equipped with an integrating sphere coated with BaSO_4_ as a reference. Samples were diluted with BaSO_4_ (30 mg sample mixed with 120 mg BaSO_4_).

#### Transmission Electron Microscopy (TEM)

The morphology and particle size distribution of the as-prepared and reduced catalysts were investigated by TEM with an FEI Titan Cryo-TEM instrument operating at an acceleration voltage of 300 kV. An appropriate amount of finely ground material was ultrasonically dispersed in analytical-grade absolute ethanol before deposition on holey Cu TEM grids.

Scanning transmission electron microscopy, combined with energy-dispersive X-ray analysis (STEM-EDX), was performed to determine the nanoscale distribution of elements in the samples. These measurements were performed on a FEI-cubed Cs-corrected Titan instrument operating at an acceleration voltage of 300 kV. The Co-CeO_2_ samples were reduced at 300 °C in a flow of 20 vol % H_2_ in He for 4 h, followed by passivation at room temperature in a flow of 2 vol % O_2_ in He for 1 h. The passivated samples were crushed and sonicated in analytical-grade absolute ethanol before deposition on holey Cu TEM grids.

#### Temperature-Programmed Reduction (H_2_-TPR)

H_2_-TPR was used to study the reduction of the samples with a Micromeritics AutoChem II instrument. Typically, approximately 100 mg of sample was loaded into a quartz U-tube between two layers of quartz wool. Before H_2_-TPR, the sample was treated at 350 °C for 1 h in a 50 mL/min flow of 5 vol % O_2_ in He. TPR profiles were recorded while heating the sample from 40 to 700 °C at a rate of 10 °C/min in a 50 mL/min flow of 4 vol % H_2_ in He. The H_2_ consumption was measured by a thermal conductivity detector (TCD), which was calibrated using an AgO reference.

#### CO and H_2_ Chemisorption

CO and H_2_ chemisorption measurements were performed with a Micromeritics ASAP2010C instrument. Typically, approximately 100 mg of sample was loaded into a quartz U-tube between two layers of quartz wool. Before chemisorption measurements, the catalyst was reduced in a H_2_ flow at 300 °C by heating to this temperature at a rate of 10 °C/min, followed by an isothermal dwell of 4 h. After evacuation at 320 °C for 1 h, CO and H_2_ adsorption isotherms­(double) were recorded at 35 and 150 °C, respectively.

#### IR Spectroscopy

IR spectra were recorded on a Bruker Vertex 70v FTIR spectrometer equipped with a DTGS detector. The experiments were performed in situ by using a home-built environmental transmission IR cell. Self-supporting pellets were prepared by pressing ∼ 10 mg of the sample into a disc with a diameter of 13 mm. Each spectrum was collected by averaging 32 scans with a resolution of 2 cm ^–1^ in the 4000–1000 cm ^–1^ range.

For CO IR spectroscopy, the sample was first reduced in a flow of 20 vol % H_2_ in He at 300 °C (rate 10 °C/min) for 4 h. After outgassing at 300 °C in vacuum and cooling to 50 °C, IR spectra were recorded as a function of the CO partial pressure in the 0–10 mbar range. CO IR measurements were also carried out at liquid N_2_ temperatures. For these measurements, the same reduction procedure was followed. After outgassing, the sample was cooled by liquid N_2_. The sample temperature was approximately −168 °C. IR spectra were recorded as a function of CO partial pressure in the 0–10 mbar range. As-prepared samples were also investigated by CO IR spectroscopy at liquid N_2_ temperature. For this purpose, the samples were evacuated at 50 °C for 1 h, before cooling to liquid N_2_ temperature. CO_2_ IR spectra were recorded at 50 °C after a similar pretreatment procedure as described for the CO IR spectroscopy measurement. The CO_2_ IR spectra were obtained as a function of CO_2_ partial pressure in the 0–10 mbar range. All spectra were background subtracted, and the intensity was normalized to the weight of the pellet.

#### X-ray Photoelectron Spectroscopy (XPS)

The surface chemical properties of the as-prepared catalysts were studied with a K-Alpha XPS instrument (Thermo Scientific) equipped with an aluminum anode (Al Kα = 1486.68 eV) monochromatized X-ray source. Finely ground samples were placed on double-sided carbon tape. All spectra were acquired using a flood gun to compensate for surface charging. A pass energy of 40 eV was used for region scans with a step size of 0.1 eV and a dwell time of 0.5 s. The spectra were analyzed using the CasaXPS software (version 3.2.23). Energy calibration was performed using the same procedure described in the NAP-XPS description.

#### Near-Ambient Pressure X-ray Photoelectron Spectroscopy (NAP-XPS)

XPS spectra were recorded in situ during the reduction of the as-prepared catalysts using a SPECS NAP-XPS system. Core-line spectra were acquired using monochromatized Al Kα radiation (1486.6 eV) generated by an Al anode (SPECS XR-50) operated at 50 W. The differential pumping system of the electron analyzer (SPECS Phoibos NAP-150) enables normal emission XPS measurements in the presence of gases, with pressures of up to ∼ approximately 20 mbar. The catalyst powder was pelletized into a disc of 12 mm in diameter, which was then fixed onto a stainless-steel sample holder. Reduction was performed in a flow of 1 mL/min H_2_ and 2 mL/min Ar. High-purity gases (99.999%) were used. The total pressure in the NAP reaction cell was kept at 3 mbar using an electronic back-pressure regulator. A typical experiment consisted of heating the sample in the reduction mixture to 550 °C while recording XPS spectra during isothermal dwells at 50 °C intervals. The total acquisition time of the XPS spectra, which included a survey scan and O 1s, C 1s, Ce 3d, and Co 2p_3/2_ spectra, was approximately 2–3 h. A pass energy of 40 eV was used with a dwell time of 0.5 s and a step size of 0.1 eV. The U”’ (Ce^4+^) component of the Ce 3d line with a characteristic position of 916.7 eV was used to correct the binding energies of the Co 2p_3/2_ and Ce 3d regions.^40,41^ A standard procedure involving Shirley background subtraction and atomic sensitivity factors was applied for data processing. Spectral lines were fitted using the CasaXPS software (version 3.2.23) with a symmetric pseudo-Voigt function (GL, 30). The main metallic component of Co was fitted with the asymmetric LA (1.2,5,5) line shape. The Ce 3d line was fitted according to a model described in the literature^42,43^

#### Quasi In Situ X-ray Photoelectron Spectroscopy (XPS)

The surface chemical properties of the reduced and deactivated catalysts were studied using a Kratos AXIS Ultra 600 equipped with a monochromatic X-ray source (Al Kα = 1486.68 eV). Self-supporting pellets were prepared by pressing approximately 40 mg of the sample into a disk with a diameter of 13 mm. Pretreatment of catalysts was carried out in a high-temperature reaction cell (Kratos, WX-530), allowing vacuum sample transfer into the analysis chamber. The samples were reduced in 20 vol % H_2_ in Ar at a flow rate of 50 mL/min at 300 °C for 4 h, with a heating rate of 10 °C/min and ambient pressure. Then, the sample was cooled to 100 °C in the pretreatment mixture, and the reaction cell was evacuated to a pressure of less than 10^–8^ mbar. Then, the sample was transferred to the XPS analysis chamber. A pass energy of 40 eV was typically used for region scans with a step size of 0.1 eV and a dwell time of 0.5 s. Energy calibration and fitting of Ce 3d and Co 2p were performed using the same procedure described in the XPS section.

#### X-ray absorption spectroscopy (XAS)

Extended X-ray absorption fine structure (EXAFS) measurements at the Co K-edge (7.7 keV) were carried out in fluorescence mode at the BL22 beamline of ALBA (Spain). The Co_3_O_4_ and CoO references were measured in transmission mode. Energy calibration was performed using a Co foil (E0 = 7.709 keV). Energy selection was achieved with a Si (111) monochromator. The EXAFS data was background-subtracted and normalized. These operations and further EXAFS fitting analysis were carried out using the Demeter package (Athena/Artemis software).^44,45^ Scattering paths were calculated using the FEFF6 code based on Co_3_O_4_, Co metal, and CeO_2_ crystal structures. A Co–O–Ce single scattering path was modeled by substituting a Co atom with Ce in the Co_3_O_4_ structure. The energy shift (E_0_), the distance change (ΔR), the coordination number (CN), and the Debye – Waller factor (σ^2^) were fitted. The amplitude reduction factors (S_0_^2^) were determined by fitting the EXAFS data of the Co foil. The amplitude reduction factors were fixed during the fitting of other parameters. Fourier-transformed EXAFS is plotted as *k* ^3^-weighted data without phase correction.

### Catalytic Activity Measurements

#### CO_2_ Hydrogenation

The catalytic performance of CeO_2_ and Co-CeO_2_ samples in CO_2_ hydrogenation was carried out in a down-flow stainless-steel reactor with an internal diameter of 4 mm. The reaction pressure was atmospheric, and the temperature ranged from 200 to 300 °C. The samples were pressed, crushed, and sieved to a fraction of 125–250 μm. Typically, the reactor was filled with 50 mg catalyst diluted with 200 mg of SiC of the same sieve fraction. Before the reaction, the catalyst was reduced in a flow of 100 mL/min of 20 vol % H_2_ in He while heating from room temperature to 300 °C at a rate of 10 °C/min, followed by an isothermal dwell for 4 h. The reduced catalyst was cooled to the initial reaction temperature of 200 °C under the same flow conditions. The reaction was started by replacing the reduction gas mixture with a flow of 50 mL/min of 60 vol % H_2_, 15 vol % CO_2_, and 25 vol % Ar (CO_2_:H_2_ ratio = 1:4). The temperature was increased in steps of 25 °C using a rate of 5 °C/min. At each isothermal dwell of 160 min, the effluent gas was sampled and analyzed by an online gas chromatograph (Shimadzu, GC-2014) equipped with RT-Q-Bond (FID) and Shincarbon ST 80/100 (TCD) analysis sections. CO_2_ conversion, carbon product selectivity, and product formation rates were calculated as follows:

$$X\left(CO_{2}\right)=1-\frac{F{\left(CO\right)}_{out}+F{\left(CH_{4}\right)}_{out}+F{\left(CH_{3}OH\right)}_{out}+xF{\left(C_{x}H_{y}\right)}_{out}}{F{\left(CO_{2}\right)}_{out}+F{\left(CO\right)}_{out}+F{\left(CH_{4}\right)}_{out}+F{\left(CH_{3}OH\right)}_{out}+xF{\left(C_{x}H_{y}\right)}_{out}}$$ 1

$$S\left(product\right)=\frac{F{\left(product\right)}_{out}}{F{\left(CO\right)}_{out}+F{\left(CH_{4}\right)}_{out}+F{\left(CH_{3}OH\right)}_{out}+xF{\left(C_{x}H_{y}\right)}_{out}}$$ 2

where C_*x*_H_*y*_ represents hydrocarbons with more than one carbon atom formed during the reaction. *F* stands for the volumetric flow rate determined from the concentration measured by gas chromatography. Ar was used as an internal standard. The FID and TCD response factors were determined using gas calibration mixtures.

The reaction rate (r_CO2_ in mol_CO2_×mol_Co_^–1^ × s^–1^) was calculated and normalized to the Co content in the following manner:

$$r_{CO2}=\frac{X{\left({CO}_{2}\right)}^{*}F{\left(CO_{2}\right)}_{in}}{{n_{Co}}^{*}V_{m}}$$ 3

where *F (CO*_2_ *)*_*in*_ is the known CO_2_ volumetric flow rate at the reactor inlet and *V*_m_ is the molar volume of an ideal gas at standard temperature and pressure. The rates of CH_4_ and CO were calculated in a similar manner.

## Results

### Catalyst Characterization

CoFSP and CeFSP samples were prepared using FSP. The most important physicochemical properties of the CoFSP samples are listed in Table 1. The Co contents of the samples determined by ICP-OES elemental analysis agree reasonably with the targeted values. Synchrotron XRD patterns of the as-prepared CoFSP samples are shown in Figure 1a. The dominant crystalline phase is fluorite CeO_2_. At a Co content of 10 mol % and higher, additional diffraction lines belong to Co_3_O_4_ (Figure 1b). The CeO_2_ lattice parameter (a_CeO2_) and CeO_2_ particle size (d_CeO2_) were estimated by Rietveld refinement (Table 1). The CeO_2_ lattice parameter was smaller for the CoFSP samples containing less than 10 mol % Co than for the CeO_2_ reference sample prepared by FSP. This can be attributed to the substitution of Ce cations in the CeO_2_ lattice with smaller Co cations.^46^ The CeO_2_ lattice parameter of the other samples, which contain more Co, is similar to the one determined for CeFSP. The average CeO_2_ particle size, determined from the XRD data, is approximately 9 nm. There is no clear correlation between the average CeO_2_ particle size and the Co content. The specific surface areas in the 110–180 m^2^/g range exhibit a decreasing trend with increasing Co content. For the samples containing Co_3_O_4_, the Rietveld refinement showed that the Co_3_O_4_ particle size increased from 3.9 nm for 10CoFSP, 5.5 nm for 20CoFSP, to 6.6 nm for 30CoFSP.

**Figure 1:**
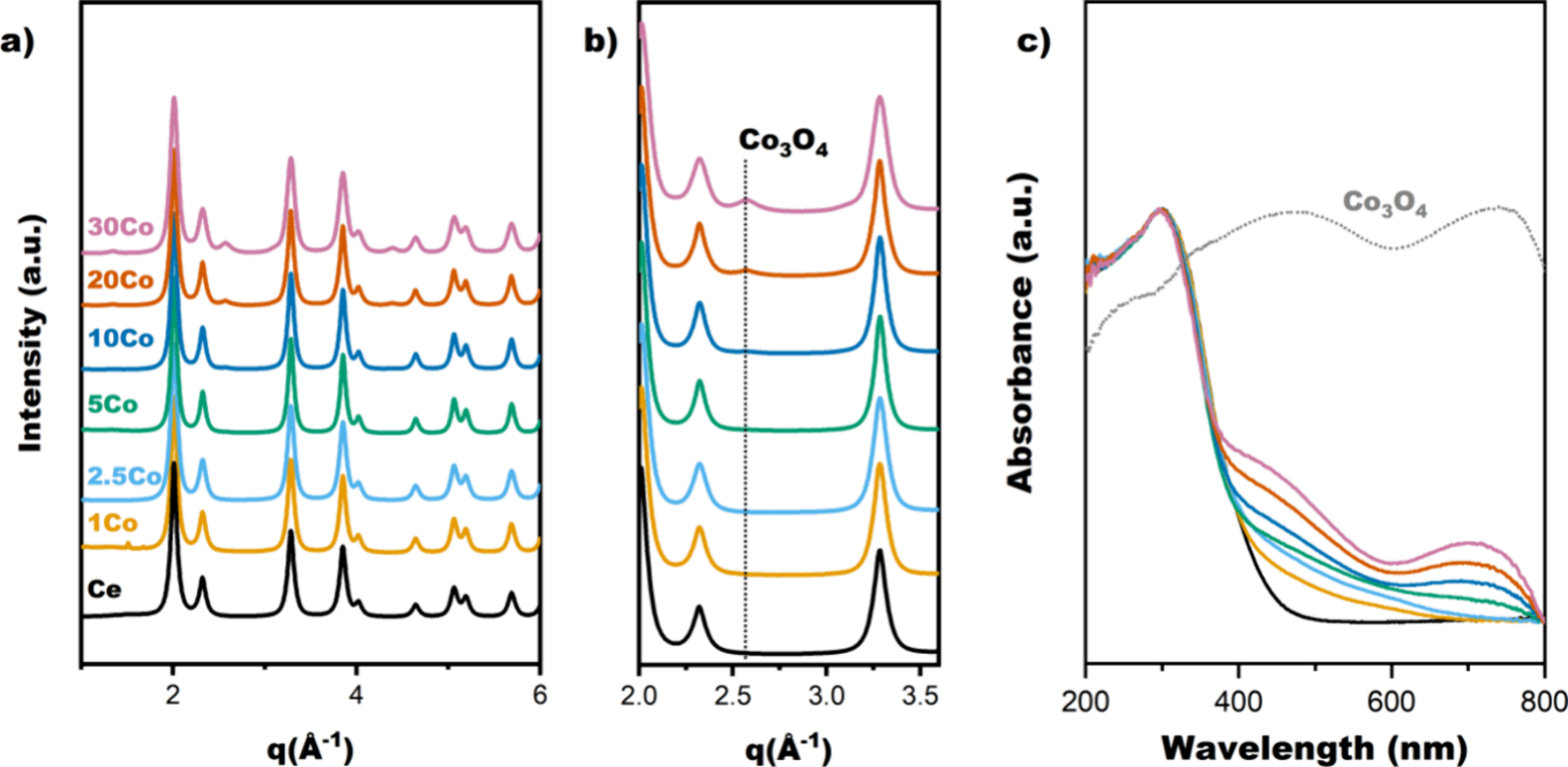
Synchrotron XRD patterns (λ = 0.182 Å) of (a) CoFSP and CeFSP with (b) a zoom in the *q* = 2.0–3.6 Å ^–1^ region, highlighting the Co_3_O_4_ (220) diffraction line (c) UV–vis spectra of CeFSP, CoFSP, and the Co_3_O_4_ reference.

**Table 1:** Physicochemical Properties of As-Prepared CoFSP and CeFSP Samples

catalyst	Co content (mol %)^a^	*S*_BET_ (m^2^/g)^b^	*d*_CeO2_ (nm)^c^	*d*_Co3O4_ (nm)^c^	*a*_CeO2_ (Å)^c^	*d*_CeO2_ (nm)^d^	Co/Ce (at.%/at.%)^e^
CeFSP	0	181	9.5 ± 0.6		5.411	5.0 ± 2.0	
1CoFSP	0.9	166	10.1 ± 0.8		5.410	4.6 ± 1.8	0.01
2.5CoFSP	2.3	166	8.3 ± 0.6		5.403	4.8 ± 1.9	0.04
5CoFSP	4.6	155	9.4 ± 0.6		5.404	4.2 ± 1.9	0.06
10CoFSP	9.5	118	9.9 ± 0.4	3.9 ± 0.4	5.410	5.0 ± 2.0	0.11
20CoFSP	18	151	10.0 ± 0.6	5.5 ± 0.4	5.411	6.0 ± 2.4	0.23
30CoFSP	27	97	8.7 ± 0.6	6.6 ± 0.4	5.411	5.9 ± 1.8	0.35

a Determined from ICP analysis.

b Determined by N_2_ physisorption on as-prepared samples.

c Particle size of CeO_2_ (d_CeO2_), particle size of Co_3_O_4_ (d_Co3O4_), and lattice parameter of CeO_2_ (a_CeO2_), determined by Rietveld refinement of synchrotron XRD on as-prepared samples.

d Determined by TEM for as-prepared samples.

e Determined by XPS for as-prepared samples.

UV–vis spectra of CeFSP and as-prepared CoFSP catalysts (Figure 1c) are characterized by bands in the 260–280 nm range, which can be assigned to CeO_2_.^47^ The formation of Co doped into CeO_2_ in samples with less than 10 mol % can be judged from the broad band in the 400–600 nm range (Figure 1c).^48^ The samples containing 10 mol % Co and more showed bands in 400–480 and 700–760 nm ranges characteristic of Co_3_O_4_. These bands represent ligand-to-metal O^2–^ - Co^2+^ and O^2–^- Co^3+^ charge transfer, respectively.^49^

Representative TEM images (Figure S2) show that the samples contain octahedrally shaped particles, irrespective of the Co content. The average particle size ranges from 4 to 6 nm, with no noticeable trend observed with the Co content.^50^ Due to the poor contrast, no clear Co-oxide particles could be distinguished. STEM-EDX maps of some of the as-prepared CoFSP samples in Figure 2 reflect the nanoscale distribution of Co. At the lowest Co content (2.5CoFSP), the maps suggest a homogeneous distribution of Co in the CeO_2_ particles. At higher Co content (5CoFSP), some Co-containing particles become visible. These particles are even more evident in the 10CoFSP sample with an average size of ∼ 4 nm.

**Figure 2:**
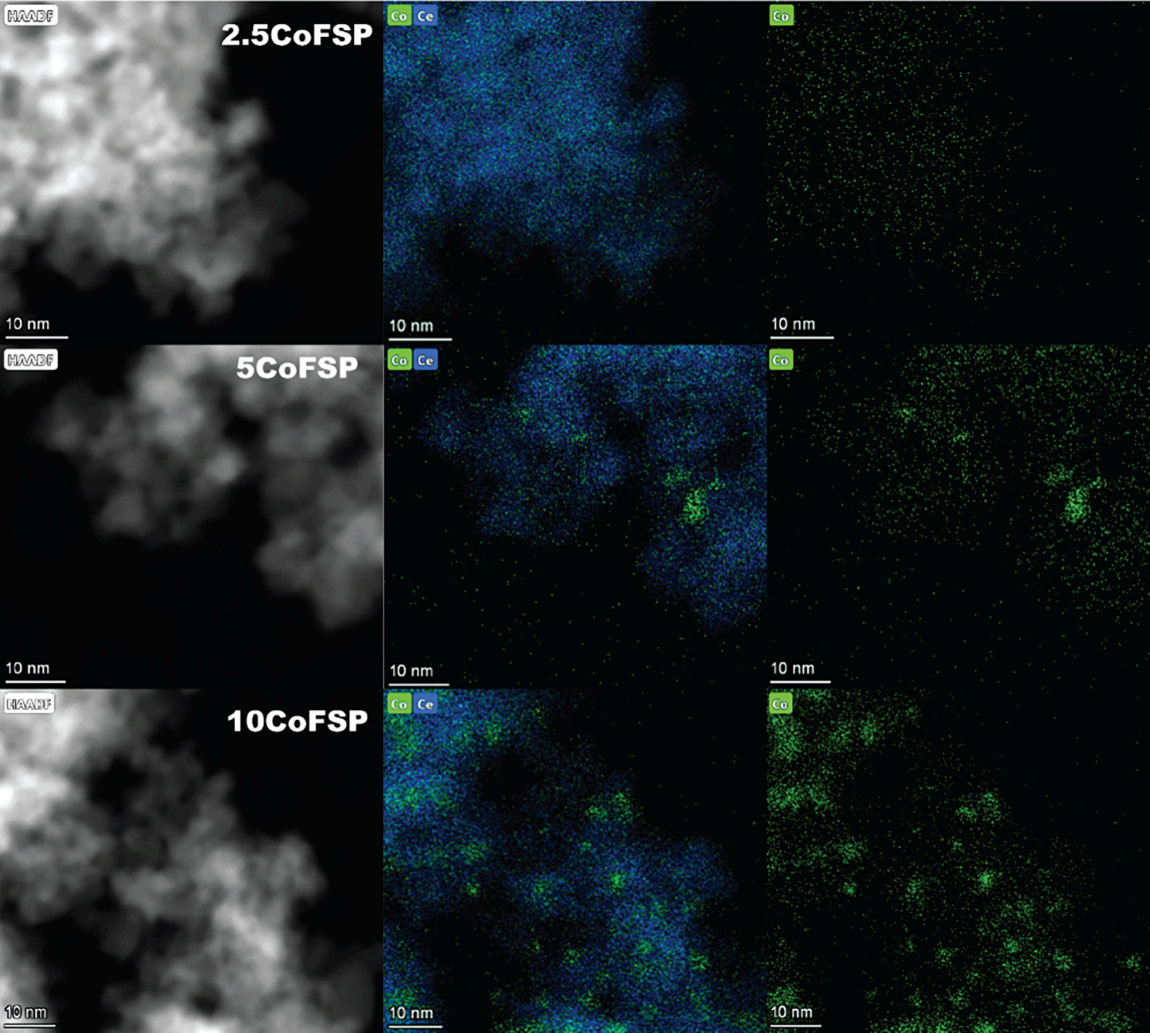
STEM-EDX images: HAADF-STEM images (1st column) and corresponding EDX elemental maps of mixed Co and Ce (2nd column), and Co (3rd column) for as-prepared CoFSP catalyst.

XPS and CO IR spectroscopy were used to probe the surface composition of the as-prepared samples. Deconvoluted XP spectra are shown in Figure S3. The XP spectra of 1CoFSP, 2.5CoFSP, and 5CoFSP catalysts can be fitted with Co^2+^ components, as reflected by the Co 2p_3/2_ peaks at binding energies of 781.0 eV, 782.4 eV, and the accompanying satellite at 786.5 eV. At Co contents above 5 mol %, the spectra can be fit by a 2p_3/2_ contribution of Co^3+^ at 778.8 eV and two Co^2+^ contributions at 780.6 and 782.4 eV.^51^ The Co^3+^ fraction for these samples is ∼ 45%, which is lower than the expected Co^3+^ fraction in Co_3_O_4_. These samples also contain Co^2+^ ions in the CeO_2_ lattice (Co-CeO_2_ solid solution) or CoO.^52^ The surface Co/Ce ratios determined by XPS increase with the Co content and are slightly higher than the bulk values, suggesting that most Co is located in the surface region. The presence of Co^2+^ was also evident from the CO IR spectra recorded at liquid N_2_ temperature (Figure S4). In addition to narrow bands at 2150–2155 cm^–1^ related to CO adsorption on Ce^4+^,^53–55^ the CO IR spectra of the CoFSP samples contain a sharp band at 2090–2100 cm^–1^ due to CO adsorption on Co^2+^.^56,57^ A band due to CO adsorption on Co^3+^, which is expected at 2180 cm^–1^,^56^ was not observed in these CO IR spectra.

STEM-EDX, XRD, XPS, and UV–vis results indicate that all CoFSP catalysts contain highly dispersed Co^2+^ at the CeO_2_ surface as CoO or doped into the CeO_2_ surface. At a Co content of 10 mol % and above, Co-oxide particles are formed, which are most likely Co_3_O_4_. Figure 3a shows the amount of Co^2+^ and Co^3+^ as a function of the Co content. The amount of Co^2+^ does not vary substantially, increasing to ∼ 4.6 mol % for 5CoFSP and then plateauing at values of ∼ 3 mol % for 10CoFSP, 20CoFSP, and 30CoFSP. Highly dispersed Co^2+^ ions in the CeO_2_ surface likely represent this nearly constant amount of Co^2+^. In contrast, the amount of Co^3+^ increases significantly with Co content, indicating the agglomeration of Co at higher concentrations as Co_3_O_4_.

**Figure 3:**
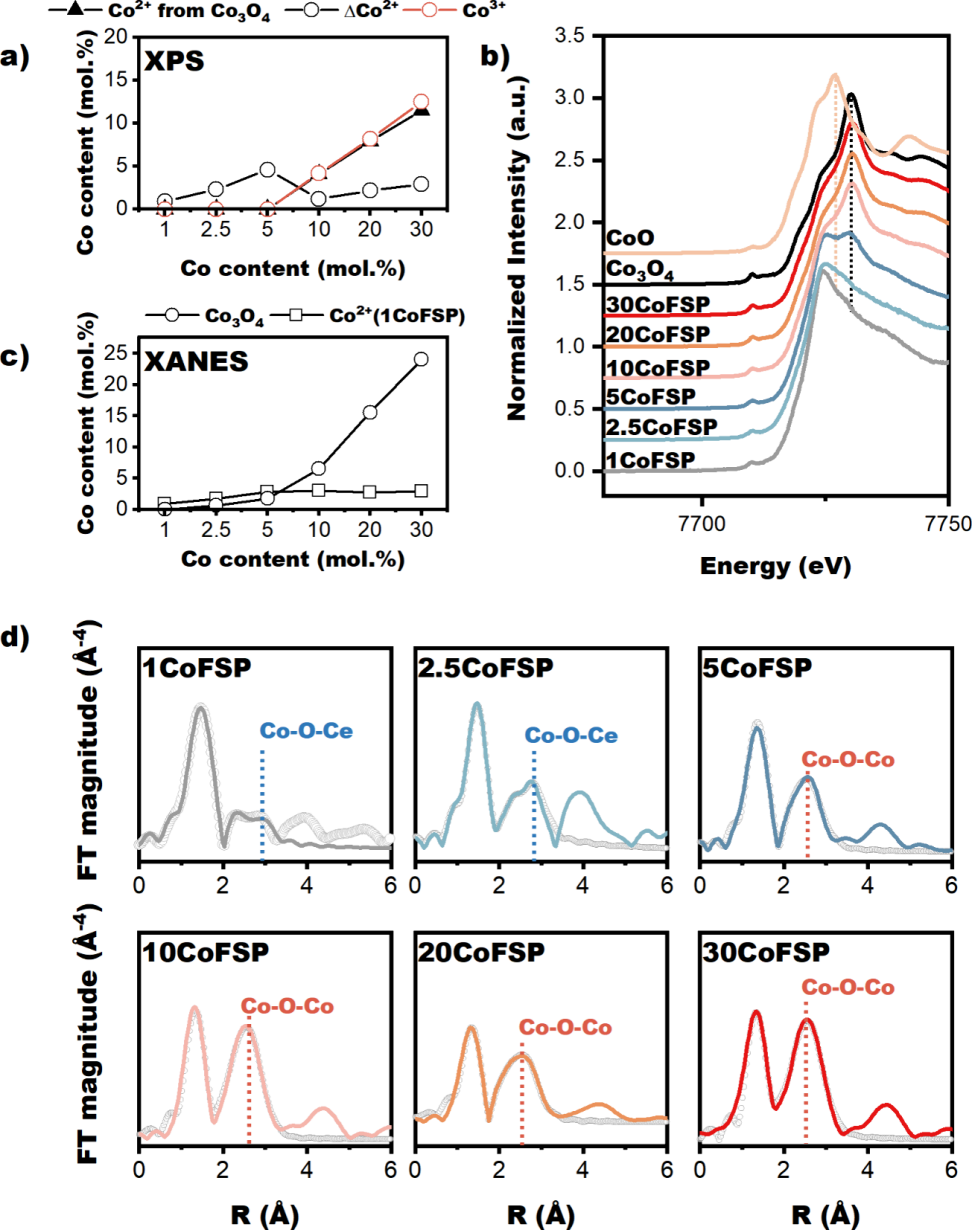
(a) Deconvolution results of Co 2p_3/2_ for as-prepared CoFSP catalysts (dark gray round, the difference between total Co^2+^ and Co^2+^, arising from Co_3_O_4_; orange round, Co^3+^; dark gray triangle, the estimated amount of Co^2+^ from Co_3_O_4_. (b) Normalized XANES spectra at the Co K-edge of the as-prepared CoFSP catalysts with a Co_3_O_4_ reference (black) and CoO (beige). (c) Amount of isolated Co^2+^ and Co_3_O_4_ as a function of the Co content, following from linear combination fitting of XANES spectra. (d) Co K-edge *k* ^3^-weighted R-space plots of the as-prepared CoFSP catalysts with the dashed lines indicating the uncorrected distances of the Co–O–Ce and Co–O–Co shells.

Normalized XANES spectra at the Co K-edge are presented in Figure 3b. The edge energy of the samples containing 10 mol % and more Co is 7722.0 eV, similar to the edge energy of the Co_3_O_4_ reference. Thus, these samples contain a significant amount of Co_3_O_4_. The edge energy of the samples containing less Co is 7714.7 eV, significantly lower than that of CoO (7716.6 eV). We speculate that this is due to the different electronic states of Co^2+^ ions substituting for Ce^4+^ in CeO_2_ and Co^2+^ in CoO. To determine the amount of Co in the CeO_2_ lattice and as Co_3_O_4_, we employed linear combination fitting of the XANES spectra. For this purpose, we prepared a 1CoFSP sample with a Co content of 1 mol % and used its XANES spectrum as the reference for isolated Co in CeO_2_. The bulk Co_3_O_4_ powder was used as the reference for Co_3_O_4_. The results in Figure 3c show that the amount of Co_3_O_4_ is small in the samples with 5 mol % or less Co and strongly increases for samples containing more Co. The amount of isolated Co^2+^ ions increases with Co content up to 5 mol % and then levels off at slightly lower values, in good agreement with the XPS findings.

We also analyzed Co K-edge EXAFS data to determine the local structure around the Co atoms. The real-space fits are provided in Figure 3d, while the fit parameters of the k^3^-weighted EXAFS are listed in Table S1. The 1CoFSP, 2.5CoFSP, and 5CoFSP samples contain Co–O (∼1.95 Å) and Co–O–Ce (∼3.21 Å) shells with coordination numbers (CNs) of 3.6 and 3.2 for 1CoFSP, 5.2 and 2.1 for 2.5CoFSP, and 4.0 and 4.1 for 5CoFSP, respectively. These distances and coordination numbers reasonably agree with the proposed structure of Co substituted for Ce^4+^ in CeO_2_.^58^ It is also seen that an increase of the Co content from 2.5 mol % to 30 mol % results in a decrease of the CNs of the Co – O shell from 5.4 to 4.0 and the Co – O – Ce shell from 3.2 to 1.8, while the CN of the Co – O – Co shell (∼2.67 Å) and Co – O – Co shell (∼3.21 Å) increase from 0.45 to 2.7 and from 1.3 to 4.9, respectively. Trend-wise, these structural changes align with the growing contribution of Co_3_O_4_, which contains Co in tetrahedral and distorted octahedral coordination, alongside the presence of highly dispersed Co^2+^ in strong interaction with CeO_2_.

### CO_2_ Hydrogenation over CeFSP and CoFSP

As mentioned above, the catalytic performance of the reduced CeFSP and CoFSP samples in CO_2_ hydrogenation was evaluated after reduction at 300 °C. The CO_2_ hydrogenation reaction was carried out at atmospheric pressure between 200 and 300 °C (Figure S5). The catalytic results will be discussed first, followed by a detailed study of the catalyst surface structures obtained after reduction at 300 °C. While CeFSP and 1CoFSP were not active in CO_2_ hydrogenation, the reduced CoFSP catalysts, containing more Co, hydrogenated CO_2_ to CO and CH_4_ (Figure 4). Small amounts of CH_3_OH, C_2_H_6_, and C_3_H_8_ were also observed at low reaction temperatures. The 2.5CoFSP catalyst exhibits high selectivity toward CO (∼79%) and the highest CH_3_OH selectivity of approximately 4% at 200 °C, accompanied by a low CO_2_ conversion of 0.2%. The CO_2_ conversion rate and the CH_4_ selectivity increase with the Co content. The 5CoFSP sample also produced a small amount of CH_3_OH, while only CO and CH_4_ were observed for the other samples with a higher Co content. The 30CoFSP sample achieved a CH_4_ selectivity of 92% at a CO_2_ conversion of 4.8% at 200 °C. Increasing the reaction temperature also led to a higher CO conversion rate and CH_4_ selectivity (Figure S5), in line with the suggestion that CO_2_ methanation follows the CO_2_ → CO → CH_4_ pathway.^37,59^

**Figure 4:**
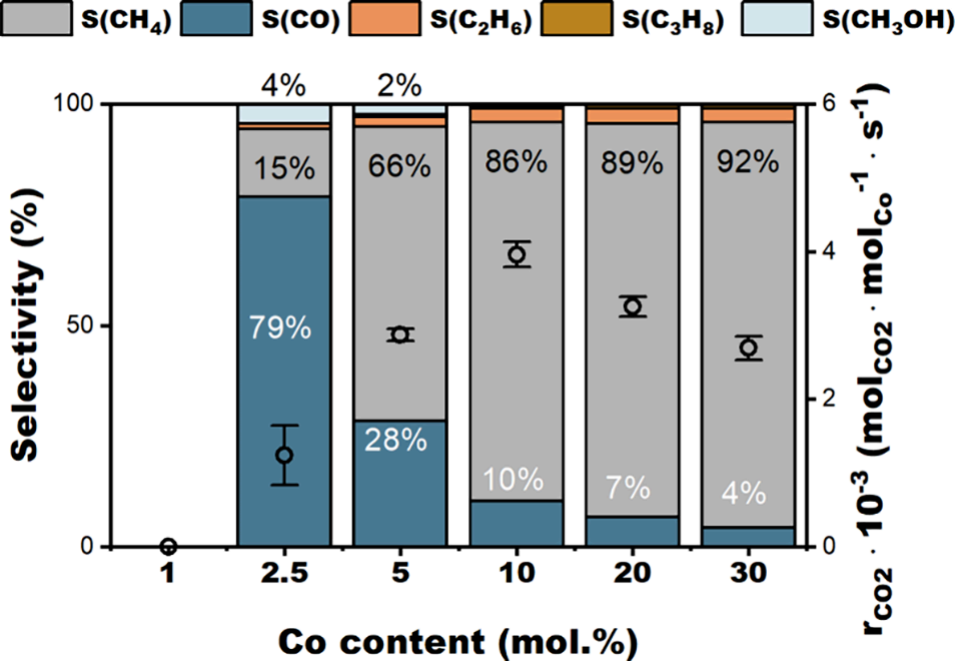
Catalytic activity and product distribution of the CoFSP catalysts reduced at 300 °C in CO_2_ hydrogenation at 200 °C. The reaction rate was normalized to the total Co content (conditions: 200 °C, 1 bar, 50 mg of sample, 15 vol% CO_2_, 60 vol% H_2_, 25 vol% Ar, 50 mL/min, 1 bar).

Figure 4 shows Co-normalized reaction rates obtained at 200 °C and differential conditions (CO_2_ conversion below 10%) as a function of the Co content. The highest reaction rate of 3.9 ± 0.2 × 10^–3^ mol_CO2_·mol_Co_^–1^ ·s^–1^ was obtained with the 10CoFSP catalyst. Normalizing the reaction rates to the number of metallic Co sites is challenging due to hydrogen spillover to the CeO_2_ support. Several mechanistic studies have emphasized that CO_2_ hydrogenation to CH_4_ requires sufficiently large metallic nanoparticles that expose step-edge sites for C–O bond dissociation in the CO intermediate.^27,60^ As small metallic Co clusters lack such sites, a high selectivity to CO has been reported for catalysts with very high Co dispersion.^52,61,62^ For Co-CeO_2_ catalysts, Co–O–Ce interfaces are also considered selective for the rWGS reaction.^52,61,62^ On the other hand, the work of Parastaev showed that very small Co clusters stabilized on CoO can catalyze CO_2_ methanation with high reaction rates.^27^ The catalysts under study here contain (i) isolated Co^2+^ in the CeO_2_ surface, which cannot be reduced at 300 °C and likely catalyze the conversion of CO_2_ to CO, and (ii) Co metal nanoparticles, whose amount and size increase with Co content, resulting in an increasing CO_2_ methanation activity. The 2.5CoFSP sample with the lowest Co content shows the highest CO formation rate and the lowest CH_4_ formation rate, which is due to the predominance of Co^2+^ in this sample. We speculate that such highly dispersed Co – O – Ce species catalyze CO formation, while a very small amount of metallic Co is responsible for CH_4_ formation. CH_4_ formation may occur on small Co clusters in close contact with Co^2+^ sites in a configuration resembling that of Co-CoO interfaces suggested by Parastaev et al.^27^ or on a small number of larger Co particles. The former Co^0^-Co^2+^–O-Ce sites might also play a role in the hydrogenation of CO_2_ to CH_3_OH. With increasing Co content, the reduced catalysts contain an increasing amount of metallic Co nanoparticles at a nearly constant amount of Co^2+^. While it is likely that the rate of CO formation on the latter sites does not vary much with the Co content, CO will be further converted to CH_4_ on the metallic Co nanoparticles. The metallic Co nanoparticles provide sites for the conventional hydrogenation of CO_2_ to CH_4_ via CO intermediate. Moreover, CH_3_OH will likely be decomposed on metallic Co, which reasonably explains the absence of CH_3_OH among the reaction products for the other catalysts. While the CO formation rate decreases with an increasing amount of metallic Co, the CH_4_ formation rate goes through a maximum for the 10CoFSP sample. We speculate that this is due to the growing size of Co nanoparticles, although we cannot exclude a role of the interface between Co nanoparticles and CeO_2_ in CO_2_ methanation. This interface of Co nanoparticles with the support will decrease with increasing Co nanoparticle size. To understand the structure sensitivity of this set of catalysts, we investigated their structure after reduction at 300 °C.

### Characterization of Co–Ce Synergy

Next, we studied the reduction behavior of these samples. Figure 5a shows the weight-normalized H_2_-TPR profiles for the CoFSP and CeFSP samples. The surface of CeO_2_ can already be reduced at relatively low temperatures in the presence of metals that can activate H_2_. Bulk reduction of CeO_2_ occurs at temperatures above 700 °C.^63,64^ CeFSP exhibits a single reduction feature at 580 °C, attributed to the reduction of surface Ce^4+^ to Ce^3+^.^65–68^ All CoFSP catalysts exhibit a low-temperature peak at 200 °C due to the reduction of surface-adsorbed oxygen species.^69,70^ The H_2_-TPR profiles of 1CoFSP and 2.5CoFSP are characterized by a main reduction peak at 350 °C. The broad reduction peak at 350 °C can be assigned to the reduction of CoO in strong interaction with CeO_2_ or Co in a Co-CeO_2_ solid solution.^68^ This interpretation is consistent with the XAS, XRD, and XPS findings, indicating the predominance of Co^2+^ species at low Co content. Increasing the Co content from 1 mol % to 2.5 mol % shifts the main reduction peak to lower temperatures. This suggests that Co in 1CoFSP is more difficult to reduce and may be due to the reduction of Co at the Co–O–Ce interface, typically observed at 400–600 °C.^68^ At a Co content of 5 mol % and above, the H_2_-TPR profiles show two peaks at 300 and 425 °C, owing to the reduction of Co_3_O_4_ to CoO and CoO to Co, respectively.^71–73^ The corresponding H_2_ consumption and estimated CeO_2_ reduction degree are shown in Table S2. The excess of consumed H_2_ is defined as the difference between the total H_2_ consumed and H_2_ required to reduce CoO/Co_3_O_4_ completely. This excess is substantial for all catalysts and varies slightly with the Co content (Figure 5b). Thus, the formation of metallic Co facilitates hydrogen spillover and partial reduction of the CeO_2_ surface.

**Figure 5:**
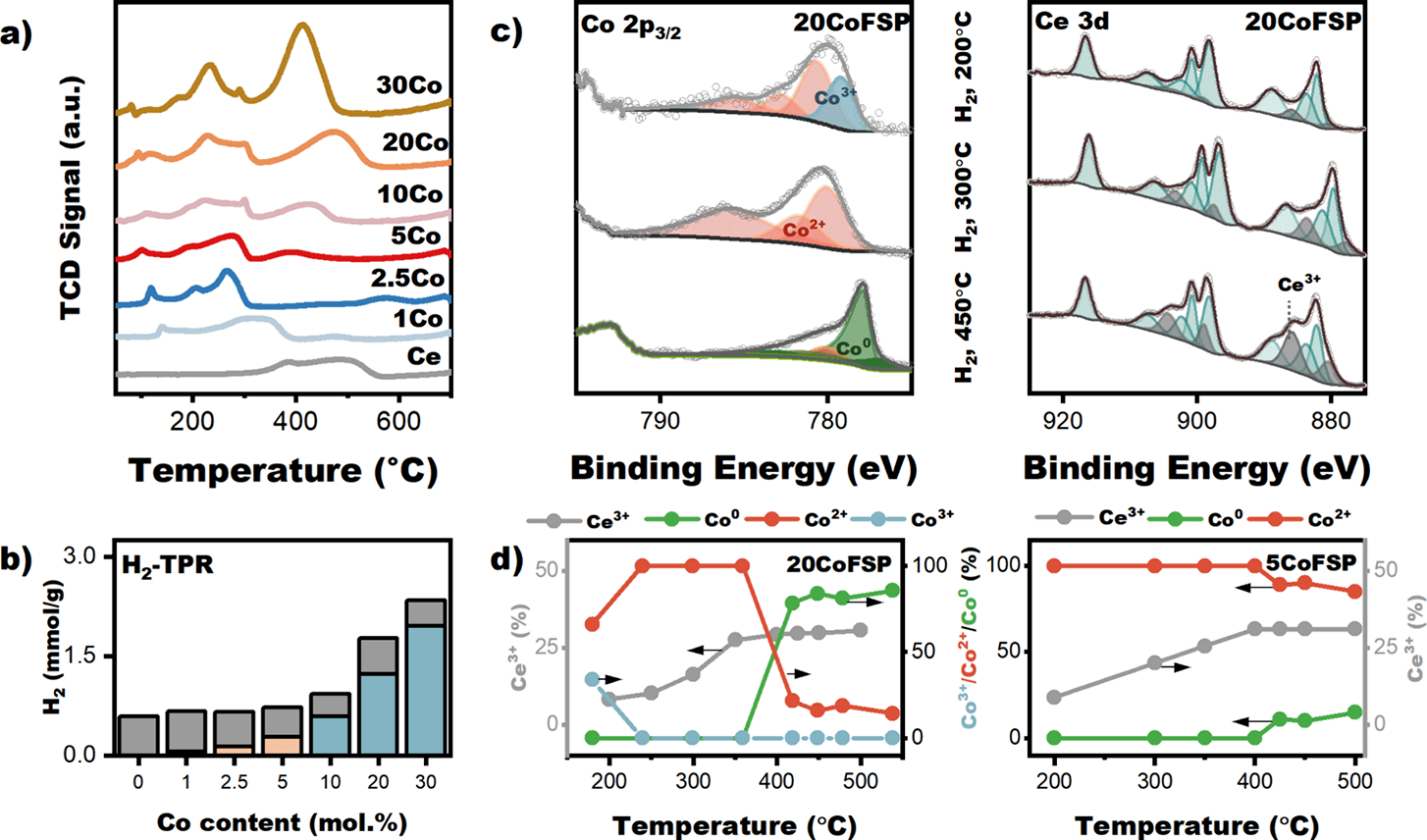
(a) Weight-normalized TCD signal of TPR profiles of CeFSP support and CoFSP catalysts (conditions: 4 vol.% H_2_, 50 mL/min). (b) Amount of H_2_ consumed during TPR experiments (gray, excess H_2_; light orange, H_2_ required for complete CoO reduction; light blue, H_2_ required for complete Co_3_O_4_ reduction. (c) Deconvoluted Co 2p_3/2_ (left) and Ce 3d (right) spectra obtained during H_2_-TPR of 20CoFSP followed by NAP-XPS (conditions: 33.3 vol.% H_2_ in Ar, 3 mbar, 200–500 °CC). (d) Co^3+^ (light blue), Co^2+^ (orange), Co^0^ (green) and Ce^3+^ (gray) contributions for 20CoFSP and 5CoFSP (conditions: 33.3 vol.% H_2_ in Ar, 3 mbar, 200–500 °C; spectra in Figure 5c and Figure S6).

To facilitate the assignment of the reduction peaks in the H_2_-TPR profiles, we investigated the reduction of the 5CoFSP and 20CoFSP samples by in situ NAP-XPS. Figure 5c shows the deconvoluted Co 2p_3/2_ and Ce 3d spectra of 20CoFSP obtained during a H_2_-TPR treatment in NAP-XPS. The corresponding results for 5CoFSP are shown in Figure S6. The most significant changes in the XP spectra occurred in the 200–400 °C range. Figure 5d plots the Co speciation (Co^3+^, Co^2+^, and Co^0^ fractions) and the Ce^3+^ fraction as a function of the reduction temperature for both samples. The contribution of Ce^3+^ increased gradually to ∼ 30% between 200 and 400 °C, already before the observation of Co^0^. The Co^0^ fraction increased to ∼ 19% (representing an amount of ∼ 1 mol % Co) for 5CoFSP and 80% (representing ∼ 14 mol % Co) for 20CoFSP, confirming the substantial difference in Co reduction degree.

The 20CoFSP sample exhibited conventional reduction behavior, where Co_3_O_4_ first transformed to CoO in the 200–250 °C range, followed by Co^0^ formation above 350 °C. In contrast, for 5CoFSP, this transformation started above 400 °C (Figure 5d). The observed shift of Co^2+^-to-Co^0^ reduction to a higher temperature in 5CoFSP compared to 20CoFSP can be attributed to the smaller size of CoO clusters in 5CoFSP,^74^ which likely results in stronger Co–CeO_2_ interactions for these smaller CoO clusters.^68^ Based on the XPS analysis after reduction at 500 °C, we determined that the reduced samples contain almost the same amount of Co^2+^, i.e., 3.7 mol % for 5CoFSP and 3.8 mol % for 20CoFSP. Likely, these species are highly dispersed Co^2+^ ions in strong interaction with the CeO_2_ support, which cannot be reduced at 500 °C. The high degree of reduction of the CeO_2_ surface observed when the samples are reduced above 400 °C aligns with the formation of Co^0^, supporting the notion of H spillover from metallic Co to CeO_2_, which implies the formation of oxygen vacancies.^75^ Based on this data, we can attribute the broad feature above 300 °C in the TPR profiles of 5CoFSP and 20CoFSP to the reduction of Co^2+^ to metallic Co and partial reduction of CeO_2_.

We employed quasi-in situ XPS to study the surface composition and degree of reduction of the reduced CoFSP samples. The samples were reduced in the reactor chamber of a Kratos XPS system. The reduction was carried out at 300 °C for 4 h at atmospheric pressure in a flow of 20 vol % H_2_ in He. The resulting Co 2p_3/2_ and Ce 3d XP spectra, along with their fits, are shown in Figure 6a, and the fit results are presented in Table S3. The fraction of metallic Co in the reduced samples was 9% for 2.5CoFSP, 14% for 5CoFSP, 52% for 10CoFSP, and 74% for 20CoFSP. The amount of highly dispersed Co^2+^ in the reduced samples was nearly constant, i.e., ∼ 4 mol % (Figure 6b). The contribution of metallic Co increased with Co content, indicating that segregated CoO and Co_3_O_4_ particles in the as-prepared samples are easier to reduce than Co incorporated into CeO_2_.^76^ If we assume that XPS can probe all Co, the amount of reduced Co atoms increased from 0.2 mol % for 2.5CoFSP to 13.5 mol % for 20CoFSP. Deconvolution of the Ce 3d XP spectra shows that reduction increased the Ce^3+^ fraction from ∼ 8% in the as-prepared samples to ∼ 33% in the reduced catalysts. The XPS Co/Ce surface ratios in the reduced CoFSP catalysts were lower than those in the as-prepared catalysts (Table 1 and Table S3). This suggests that sintering of Co species occurs during the reduction step, considering that the encapsulation of Co by CeO_2_ is unlikely to occur at the low reduction temperature used.

**Figure 6:**
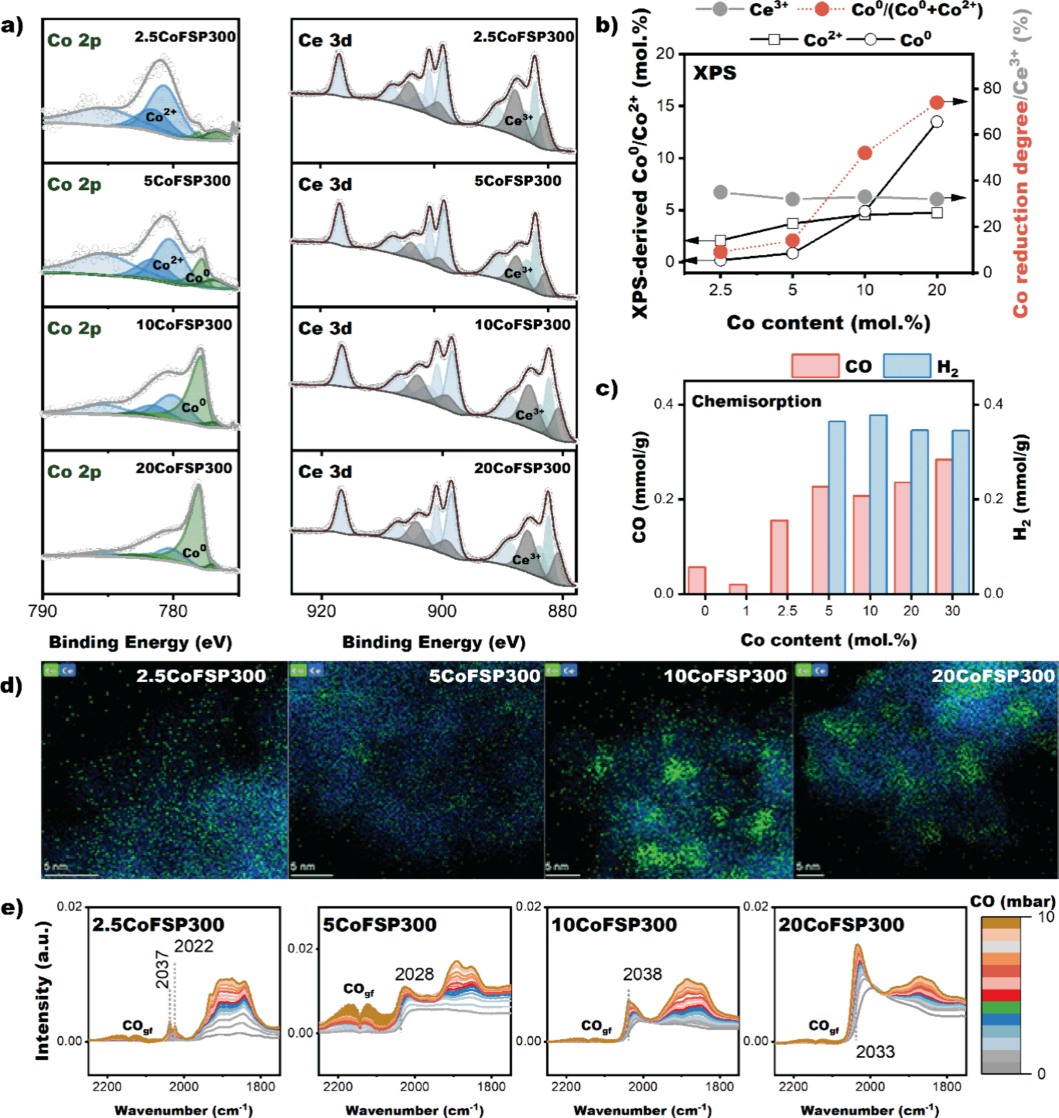
(a) Deconvolution of Co 2p_3/2_ and Ce 3d XP spectra of 2.5CoFSP, 5CoFSP, 10CoFSP, and 20CoFSP after reduction at 300 °C (conditions: 20 vol.%H_2_ in Ar, 50 mL/min, 10 °C/min, 4 h). (b) Co^2+^ (black square), Co^0^ (black round) contributions, Co reduction degree (Co^0^/(Co^0^+Co^2+^)) and Ce^3+^ fraction derived from XP spectra in panel (a). (c) Amount of H_2_ and CO chemisorbed during chemisorption measurements on CeFSP and CoFSP samples reduced at 300 °C. (d) EDX elemental maps of Co and Ce for reduced and passivated CoFSP catalyst. (e) IR spectra of the CoFSP samples reduced at 300 °C after CO adsorption at 50 °C (conditions: 1–10 mbar CO).

After reduction in H_2_ at 300 °C, substantial differences in the Co metal particle size are observed, as follows from H_2_ chemisorption (Table S3, Figure 5c, Figure S7, Note S1) and CO chemisorption (Table S3, Figure 5c, Figure S8, Note S2) measurements. At a Co content of 2.5 mol % and higher, the amount of chemisorbed CO and H_2_ increases strongly, suggesting the presence of metallic Co. The Co particle size in the reduced and passivated CoFSP catalysts was estimated from HAADF-STEM-EDX maps (Figure 6d). At low Co content (2.5CoFSP), Co remains highly dispersed on CeO_2,_ similar to the EDX maps of the as-prepared 2.5CoFSP (Figure 2). Increasing the Co content to 5 mol % led to some Co clusters with a size of ∼ 1.5 nm. The average Co particle sizes were ∼ 4.5 nm and ∼ 6.5 nm for reduced 10CoFSP and 20CoFSP, respectively. The EDX maps indicate that all the catalysts also contained highly dispersed Co.

The Co speciation in the reduced catalysts was also investigated by CO IR spectroscopy at 50 °C and liquid N_2_ temperature (Figure 6e and S8–10, Note 3–4). The assignment of the IR bands is shown in Table S4. The spectra recorded at 50 °C are characterized by linear and bridged carbonyl bands in the 2060–2000 cm^–1^ and 1970–1840 cm^–1^ ranges, respectively.^77^ The narrow carbonyl band in the 2020–2040 cm^–1^ range for 2.5CoFSP points to CO adsorption on small Co clusters.^27,62,77^ With increasing Co content, the position of the linear carbonyl band shifts to lower wavenumbers (2000 cm^–1^). With increasing CO partial pressure, the carbonyl band shifts to 2040 cm^–1^ due to lateral interactions between adsorbed CO molecules, which is typical for metallic Co nanoparticles.^78,79^ The corresponding IR spectra recorded at liquid N_2_ temperature further support the conclusion that the reduced 2.5CoFSP sample likely contains small metallic Co clusters and Co^2+^, while the reduced 5CoFSP, 10CoFSP, and 20CoFSP contain metallic Co nanoparticles, as evident from the IR-CO spectra (Figure S9–11 and Note S3). The negative band at 2090 cm^–1^ in the CO_2_ IR spectra indicates that CeO_2_ was partially reoxidized by CO_2_ in the reduced CoFSP catalysts (Figure S12–13, Note S5). Moreover, a broad band was observed in the 2110–2135 cm^–1^ range for the reduced CeO_2_-based catalysts, which is due to the ^2^F5/2 → ^2^F7/2 electronic transition of Ce^3+^.^53^ The appearance of this band upon reduction evidence the partial reduction of CeO_2_ in the CeFSP and CoFSP catalysts.^75^

The structural changes of the CoFSP catalysts and the bare CeFSP support during reduction were also investigated by in situ synchrotron XRD and PDF (Figure S14–17, Notes S6–7, Tables S5–6). No reflections related to Co-containing phases were observed on 2.5CoFSP and 5CoFSP, suggesting that the reduced Co particles were very small, while the Co nanoparticles formation is evident for 10CoFSP and 20CoFSP after reduction, which is in line with the STEM-EDX maps and IR-CO spectra. Rietveld refinement of the XRD patterns and PDF revealed an increase in the CeO_2_ unit cell parameter after reduction at 300 °C, indicative of the partial reduction of CeO_2_ and subsequent oxygen vacancies formation (Figure S15, Note S5–6; Table S5–6).

## Discussion

Combined XRD, XAS, and STEM-EDX data demonstrate that introducing Co into CeO_2_ first leads to the formation of highly dispersed Co^2+^, most likely dispersed in the CeO_2_ as a solid solution (1CoFSP and 2.5CoFSP). The substitution of Ce with Co is evident from the unit cell contraction probed by XRD. A higher Co content led to Co-oxide particles and a nearly constant amount of highly dispersed Co^2+^. The presence of Co species differing in their interaction with CeO_2_ led to different reduction behaviors, as followed from H_2_-TPR and NAP-XPS measurements. Reduction at 500 °C led to a small amount of Co^0,^ with most (∼80%) of Co remaining highly dispersed. On the other hand, the same reduction treatment led to the reduction of nearly 80% of Co in 20CoFSP, resulting in the predominance of Co metal nanoparticles. The amount of highly dispersed Co^2+^ was roughly constant in all samples, suggesting that these species are too strongly bound to CeO_2_ to be reduced under the given conditions. The presence of such uniformly distributed Co species was confirmed by STEM-EDX maps in the as-prepared and reduced samples. Thus, while highly dispersed Co – O – Ce species are not reduced, the CoO and Co_3_O_4_ particles in the as-prepared CoFSP precursor result in nanometer-sized Co particles upon reduction. Irrespective of the Co content, the surface of CeO_2_ was partially reduced, indicating the formation of oxygen vacancies for all CoFSP catalysts during reduction. Oxygen vacancies in CeO_2_ are thought to be involved in CO_2_ hydrogenation, for example, by enhancing CO_2_ adsorption.^80,81^ The 2.5CoFSP sample with the lowest Co content exhibited the highest CO formation rate and the lowest CH_4_ formation rate, likely due to the small amount of metallic Co in small clusters. We cannot exclude the fact that the highly dispersed Co – O – Ce species also contribute to CO formation.^61,82,83^ Active sites at the interface between small Co^0^ clusters and Co^2+^–O-Ce moieties might play a role in the hydrogenation of CO_2_ to CH_3_OH,^17^ which was observed for 2.5CoFSP and 5CoFSP. With increasing Co content, the reduced catalysts contain more metallic Co nanoparticles at a nearly constant amount of Co^2+^. The metallic Co nanoparticles provide sites for H_2_ dissociation and conventional hydrogenation of CO_2_ to CH_4_ via CO intermediate.^59,84,85^ These samples showed very little or no CH_3_OH formation, likely because CH_3_OH is decomposed on metallic Co. Comparison of the Co-weight normalized activity with literature data and the reference Ni-based catalysts (Figure S18-19 and Table S7) shows that the 10CoFSP catalyst is more active in CO_2_ methanation than the most active Co/CeO_2_ and Co/TiO_2_ catalysts reported, exhibiting higher CH_4_ selectivity and CO_2_ conversion in the 200–300 °C temperature range. It is speculated that the combination of small Co nanoparticles with oxygen vacancies in the CeO_2_ surface contributes to the high activity in CO_2_ methanation.

## Conclusions

This work evaluated a set of Co-CeO_2_ samples with varying Co content (1–30 mol %) prepared by FSP for their catalytic performance in CO_2_ methanation. FSP preparation of Co-CeO_2_ resulted in small CeO_2_ nanoparticles of ∼ 8 nm with a higher surface area than conventional CeO_2_. Catalysts with low Co content contain a relatively large fraction of Co^2+^ ions in strong interaction with CeO_2_, which cannot be reduced at 500 °C. The amount of such stable Co^2+^ species is nearly the same in all samples containing 5 mol % Co or more, i.e., ∼ 3.8 mol %. Catalysts containing 5 mol % Co or more contain additionally segregated Co-oxide (CoO and Co_3_O_4_) particles. These particles can be reduced to metallic Co nanoparticles at 300 °C. The highest Co-weight-normalized activity of 3.9 ± 0.2 mmol_CO2_/mol_Co_/s at a temperature of 200 °C was found for the 10CoFSP sample. The Co reduction degree of this sample is ∼ 50%, represented by ∼ 4.5 nm Co nanoparticles, with the other half of Co present as highly dispersed Co^2+^ in strong interaction with the CeO_2_ support. This sample exhibited a CH_4_ selectivity of 85% at 200 °C. A very low Co reduction degree of ∼ 10% in 2.5CoFSP with only small Co clusters as the metallic phase led to the predominant formation of CO (79%) and less CH_4_ (17%). A small amount of CH_3_OH among the reaction products is associated with the hydrogenation of CO_2_ on oxygen vacancies assisted by H_2_ dissociation on small Co clusters. Catalysts containing more and larger Co nanoparticles mainly yield CH_4_, small amounts of CO, and no CH_3_OH. The high CO_2_ methanation activity of FSP-prepared Co-CeO_2_ catalysts is linked to the synergy between relatively small metallic Co nanoparticles for CO methanation and Co^2+^–O-Ce sites, involving oxygen vacancies, for CO_2_-to-CO conversion.

## Supplementary Material



## Notes

Schematic representation of FSP synthesis and structure of the CoFSP catalysts as a function of Co content (Figure S1); TEM results of the as-prepared Co-CeO_2_ catalysts (Figure S2); additional characterization (XPS spectra and CO-IR spectra of as-prepared CeO_2_ and Co-CeO_2_ catalysts (Figures S3 and S4); fit parameters of EXAFS data at the Co K-edge (Table S1); catalyst reducibility of CeFSP and CoFSP catalysts determined by H_2_-TPR (Table S2); catalytic results of the Co–CeO_2_ catalysts (Figure S5); physicochemical properties of reduced at 300 °C CoFSP and CeFSP samples. (Table S3); XPS spectra of 5CoFSP recorded during H_2_-TPR during NAP-XPS experiment (Figure S6); H_2_ and CO chemisorption of the reduced Co-CeO_2_ catalysts (Figures S7 and S8; Notes S1 and S2; Table S3); CO- and CO_2_–IR spectra and assignment of IR bands (Figures S9–S13, Notes S3–S5, and Table S4); structural changes of CeO_2_ and Co-CeO_2_ catalysts during H_2_ reduction (XRD and PDF) (Figures S14–S17, Note S6, and Tables S5 and S6); comparison of CO_2_ hydrogenation performance over different Co-based catalysts (Figure S18; Table S7); catalytic results of the reference 10Ni/CeO_2_ and 10Ni/SiO_2_ catalysts (Figure S19; Table S7) (PDF)
