# Supplementary material for: Flame Synthesized Co–CeO2 Catalysts for CO2 Methanation
Source: ACS Catal. 2025 Jun 13;15(13):11217–31. doi: 10.1021/acscatal.5c02380 (PMC12235586; doi:10.1021/acscatal.5c02380)
Supplement: Supplementary file 1 [file cs5c02380_si_001.pdf]

Supporting information

Flame Synthesized Co – CeO_2 Catalysts for CO_2 methanation

Angelina Evtushkova, Jason M.J.J. Heinrichs, Alexander Parastaev,

Nikolay Kosinov, Emiel J. M. Hensen*

Laboratory of Inorganic Materials and Catalysis, Department of Chemical Engineering and Chemistry, Eindhoven University of Technology, 5600 MB Eindhoven, The Netherlands.

* E-mail: e.j.m.hensen@tue.nl

Table of contents Supporting information

Supplementary figures, tables, and notes 4

Flame-spray pyrolysis for synthesis of the catalysts 4

Figure S1. .... *4*

Characterization of the as-prepared samples 5

Figure S2. .... *5*

Figure S3. .... *6*

Figure S4. .... *7*

Table S1. .... *8*

CoFSP catalysts in CO_2 hydrogenation 9

Figure S5. .... *9*

Reducibility of CeFSP and CoFSP 10

Table S2. .... *10*

Figure S6. .... *11*

Table S3. .... *12*

H_2 chemisorption 13

Note S1. .... *13*

Figure S7. .... *13*

CO chemisorption 14

Note S2. .... *14*

Figure S8. .... *14*

IR adsorption 15

Table S4. .... *15*

IR-CO at 50 ℃ *16*

Note S3. .... *16*

Figure S9. .... *16*

IR-CO at Liquid N_2 temperature *17*

Note S4. .... *17*

Figure S10. .... *18*

Figure S11. .... *19*

IR-CO_2 .... *20*

Note S5. .... *20*

Figure S12. .... *20*

Figure S13. .... *21*

Structural changes of CeFSP and CoFSP during reduction (XRD and PDF) 22

Note S6. 22
Figure S14. 23
Figure S15. 24
Figure S16. 25
Figure S17. 26
Table S5. 27
Table S6. 27
Comparison of CO_2 hydrogenation performance over different catalysts 28
Figure S18. 28
Figure S19. 28
Table S7. 29
References...37

Supplementary figures, tables and notes

Flame-spray pyrolysis for synthesis of the catalysts

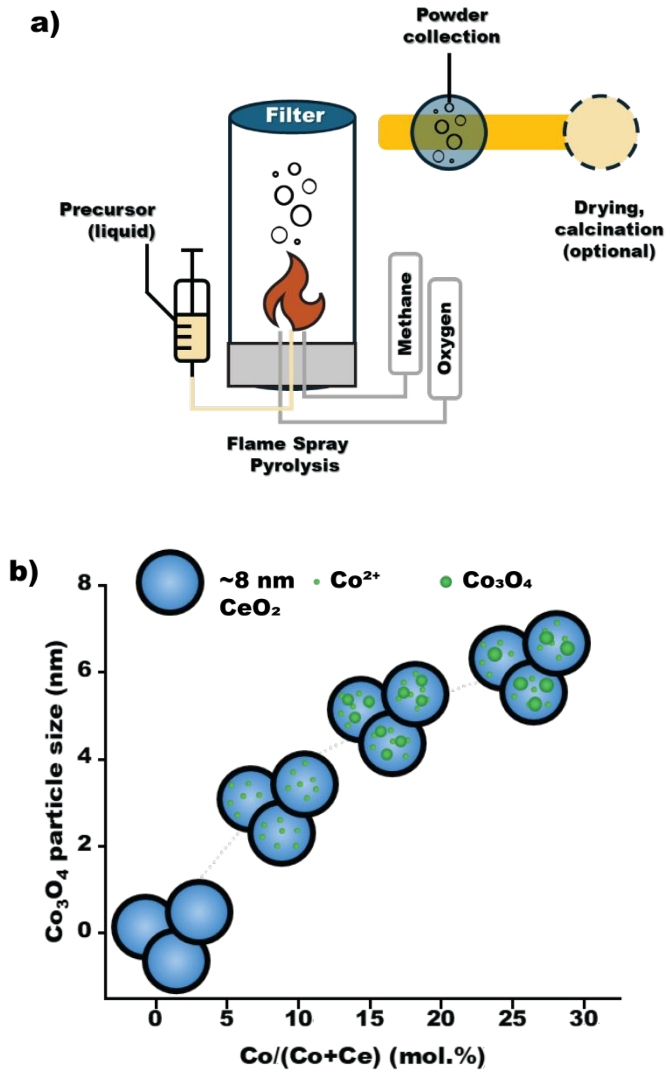

Figure S1. (a) Schematic representation of FSP synthesis. (b) Structure of the CoFSP catalysts as a function of Co content.

Characterization of the as-prepared samples

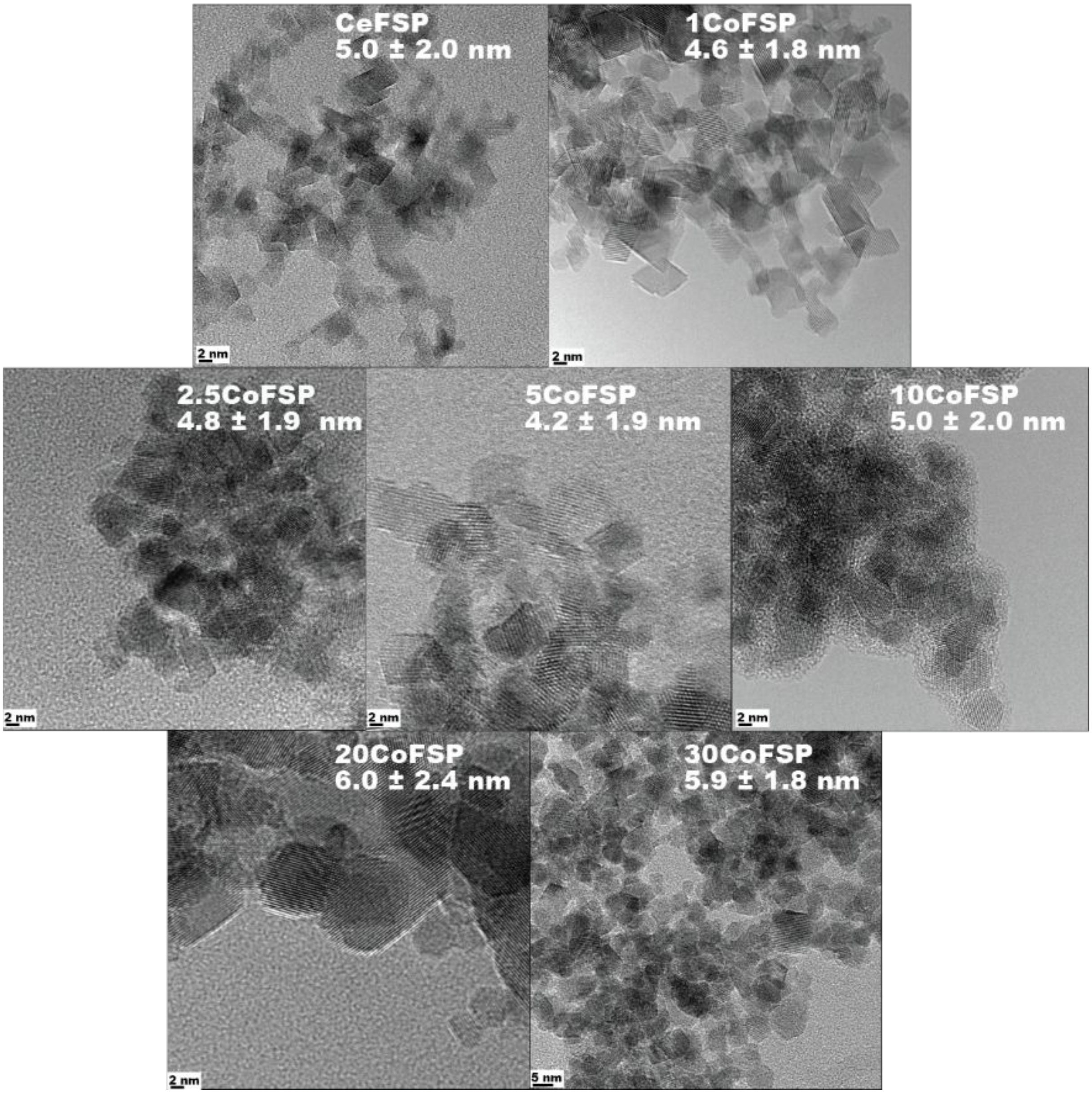

Figure S2. TEM images of as-prepared CeFSP and CoFSP catalysts with corresponding particle size estimations (scale = 2nm).

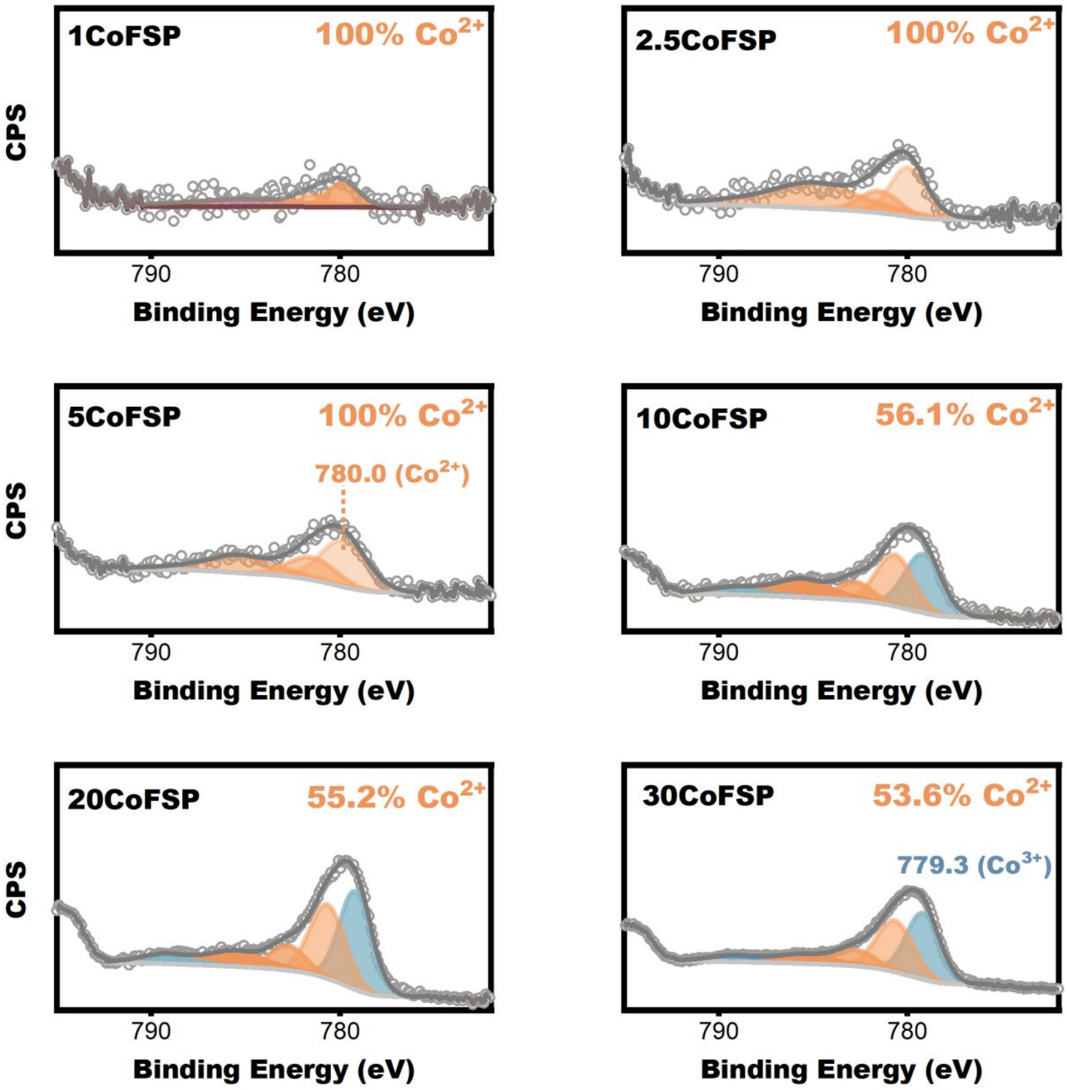

Figure S3. Deconvolution of Co $2p_{3/2}$ XP spectra of as-prepared CoFSP catalysts.

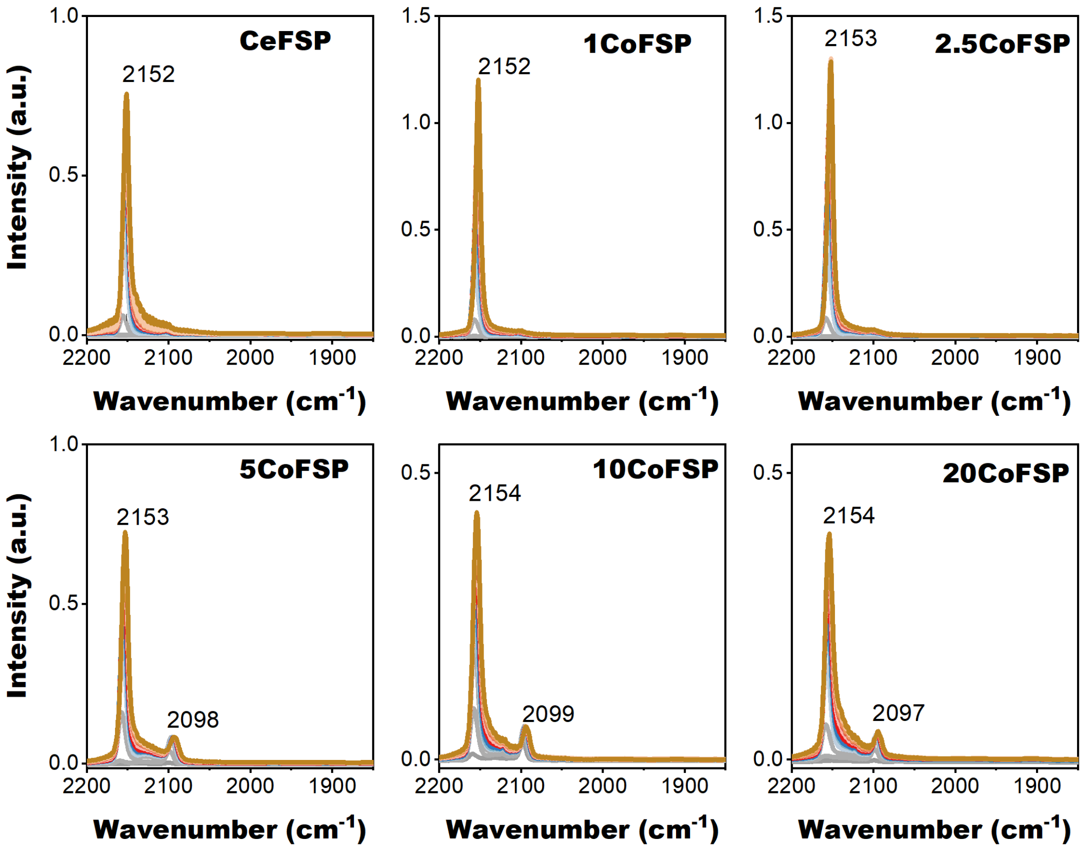

Figure S4. IR spectra of the as-prepared CeFSP and CoFSP after CO adsorption at liquid N_2 temperature (conditions: 1 – 10 mbar CO).

Table S1. Co K-edge EXAFS fit results of CoFSP samples (error margins reported in brackets).

Catalyst	Path	CN [±]	R (A) [±]	E_0 (eV)	δ^2 (A^2)	R-factor
**1CoFSP**	Co-O	3.6[0.4]	1.950[0.007]	-3.36	0.004	0.014
	Co-O-Ce	3.2[1.6]	3.214[0.022]		0.001	
	Co-Co [1]	0.45[0.4]	2.676[0.060]		0.003	
	Co-Co [2]	1.3[0.6]	3.214[0.030]		0.004	
**2.5CoFSP**	Co-Co [2]	5.2[0.3]	1.952[0.005]	-4.28	0.010	0.013
	Co-O-Ce	2.1[1.4]	2.982[0.028]		0.004	
	Co-Co [1]	0.9[0.3]	2.872[0.027]		0.004	
	Co-Co [2]	1.0[0.3]	3.211[0.026]		0.009	
**5CoFSP**	Co-O	4.0[0.3]	1.893[0.007]	-7.47	0.004	0.010
	Co-O-Ce	4.1[1.8]	3.014[0.017]		0.002	
	Co-Co [1]	1.8[0.5]	2.706[0.021]		0.002	
	Co-Co [2]	1.9[0.5]	3.355[0.017]		0.004	
**10CoFSP**	Co-O	5.2[0.3]	1.844[0.004]	-8.27	0.003	0.002
	Co-Co [1]	2.4[0.2]	2.726[0.007]		0.002	
	Co-Co [2]	4.7[0.5]	3.204[0.007]		0.004	
**20CoFSP**	Co-O	4.2[0.2]	1.848[0.008]	-5.94[1.1]	0.001	0.005
	Co-Co [1]	2.8[0.2]	2.740[0.010]		0.001	
	Co-Co [2]	4.0[0.4	3.232[0.011]		0.002	
**30CoFSP**	Co-O	4.2[0.3]	1.832[0.007]	-7.91	0.001	0.011
	Co-Co [1]	2.7[0.4]	2.724[0.009]		0.001	
	Co-Co [2]	4.9[0.6]	3.212[0.008]		0.002	

CoFSP catalysts in CO_2 hydrogenation

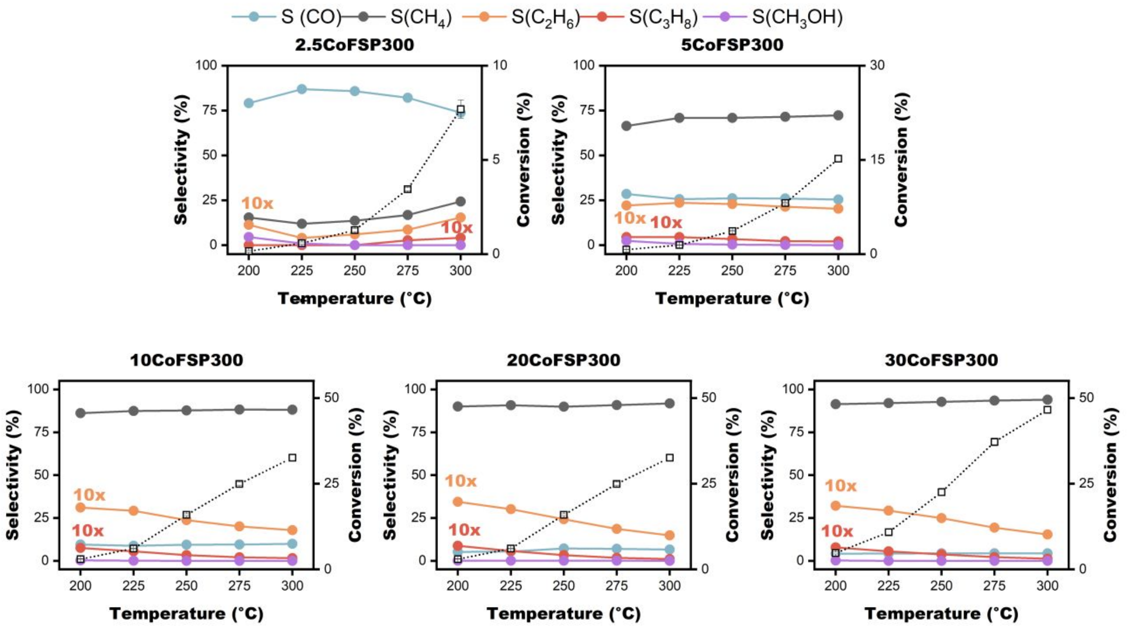

Figure S5. Catalytic performance of CoFSP catalysts reduced at 300°C in CO_2 hydrogenation as a function of temperature (conditions: 200 – 300 °C, 1 bar, 50 mg of sample, 15 vol% CO_2, 60 vol% H_2, 25 vol% Ar, 50 mL/min, 1 bar).

Reducibility of CeFSP and CoFSP

Table S2. Catalyst reducibility of CeFSP and CoFSP catalysts determined by H_2-TPR.

Catalyst	CeFSP	1CoFSP	2.5CoFSP	5CoFSP	10CoFSP	20CoFSP	30CoFSP
H_2 (mmol/g) [a]	0.59	0.78	0.66	1.12	1.45	2.11	2.89
Excess H_2 consumed (mmol/g) [b]	-	0.61	0.52	0.44	0.33	0.54	0.39

a – determined from H_2-TPR in 100 – 650 °C range, b - estimated from the difference between total H_2 consumption and H_2 required to reduce CoO/Co_3O_4 completely during H_2-TPR.

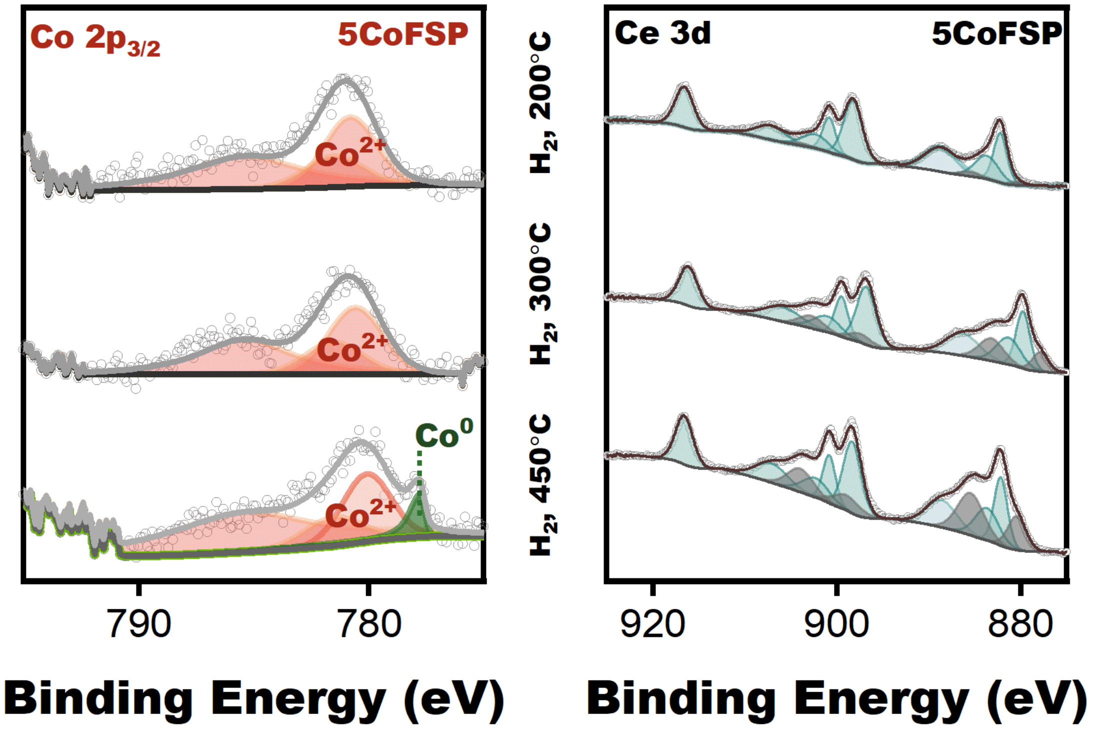

Figure S6. Deconvoluted Co $2p_{3/2}$ (left) and Ce 3d (right) spectra obtained during H_2-TPR of 5CoFSP followed by NAP-XPS (conditions: 33.3 vol.% H_2 in Ar, 3 mbar, 200 – 500 °C).

Table S3. Physicochemical properties of reduced at 300 °C CoFSP and CeFSP samples.

Catalyst	CeFSP	1CoFSP	2.5CoFSP	5CoFSP	10CoFSP	20CoFSP	30CoFSP
d_{Co} (nm)[a]		n.a.	n.a.	1.5	4.5	6.5	n.a.
H_2 chemisorbed (mmol/g) [b]	0.00	0.00	0.00	0.36	0.38	0.35	0.35
Theoretical H_2 chemisorbed (mmol/g) [c]	-	-	0.007	0.013	0.033	0.065	-
CO chemisorbed (mmol/g) [d]	0.06	0.02	0.16	0.23	0.21	0.24	0.28
Co reduction degree [e]	-	-	0.09	0.14	0.52	0.74	-
Ce^{3+} (%) [e]	-	-	35	32	33	32	-
Co/Ce (at.%/at.%)[e]	-	-	0.01	0.02	0.03	0.07	-

a – Co particle size determined by STEM-EDX on reduced at 300 °C and passivated CoFSP catalysts; b – determined by H_2 chemisorption at 150 °C on pre-reduced samples at 300 °C; c - estimated assuming a spherical shape of the Co particles and a H/Co adsorption stoichiometry of 1, the Co reduction degree, derived from XPS of reduced CoFSP catalysts, and STEM-EDX-derived Co particle sizes; d – determined by CO chemisorption at 35 °C on pre-reduced samples at 300 °C; e – Co reduction degree $Co^0/(Co^0+Co^{2+})$ and Ce^{3+} fraction determined by quasi-in situ XPS on samples reduced at 300 °C.

H_2 chemisorption

Note S1.

H_2 chemisorption measurements show that the reduced catalysts with a Co content 5 mol.% and above chemisorb H_2 irreversibly (**Table S3, Figure 6c, Figure S7**). The 1CoFSP and 2.5CoFSP catalysts do not chemisorb H_2 at 150 °C, which is likely due to the very small amount of metallic Co. The amount of chemisorbed H_2 increased with the Co content, reaching the highest value for 10CoFSP and significantly The theoretical amount of chemisorbed H_2 was estimated based on the Co particle sizes estimated from STEM-EDX maps and assuming a spherical particle shape, a H/Co adsorption stoichiometry of unity, and the Co reduction degrees determined by quasi-in situ XPS. The dispersion of a small amount of Co particles in 2.5CoFSP was assumed to be 100%. The resulting theoretical estimates of 0.013 mmol/g for 5CoFSP, 0.033 mmol/g for 10CoFSP, and 0.065 mmol/g 20CoFSP are substantially lower than the experimental H_2 chemisorption values of 0.36 mmol/g for 5CoFSP, 0.38 mmol/g for 10CoFSP and 0.35 mmol/g for 20CoFSP. The difference is caused by hydrogen spillover to CeO_2 in supported metal nanoparticle catalysts [1,2].

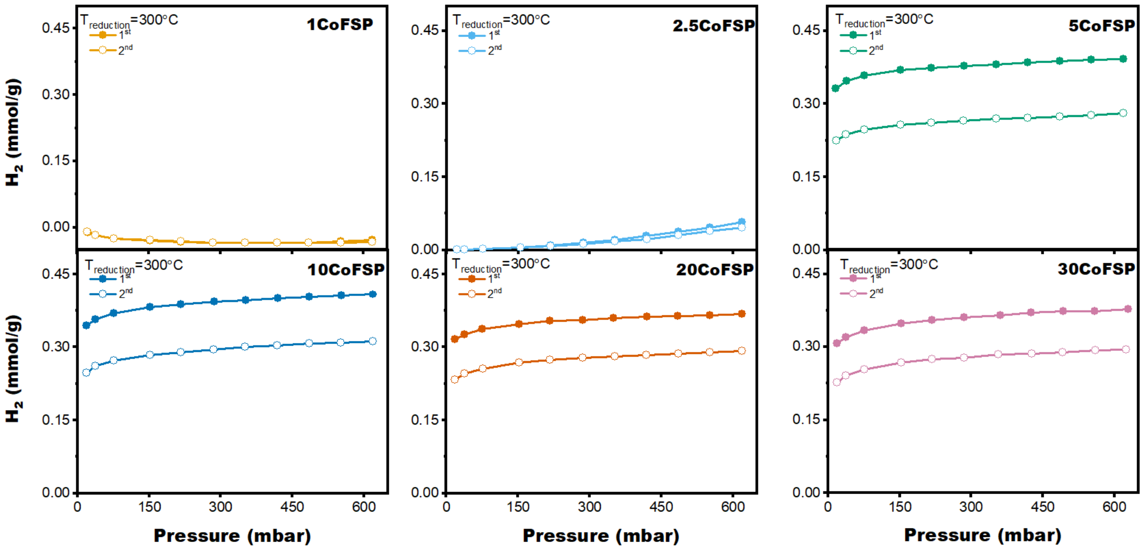

Figure S7. H_2 chemisorption at 150 °C of CoFSP reduced at 300°C for 4 h.

CO chemisorption

Note S2.

CO chemisorption revealed strong CO chemisorption on the reduced CoFSP catalysts and CeFSP (**Table S3, Figure 6c, Figure S8**). The amount of irreversibly chemisorbed CO on CeFSP indicates the presence of oxygen vacancies upon reduction [3,4]. As the H_2 chemisorption data show that more oxygen vacancies are formed in the presence of Co, a significant fraction of CO chemisorbed on the reduced Co-containing catalysts is likely due to oxygen vacancies. The amount of chemisorbed CO strongly increases at a Co content of 2.5 mol.% and higher. Although part of the chemisorbed CO is on metallic Co [5–7], CO chemisorption data cannot be used to determine the number of metallic Co sites.

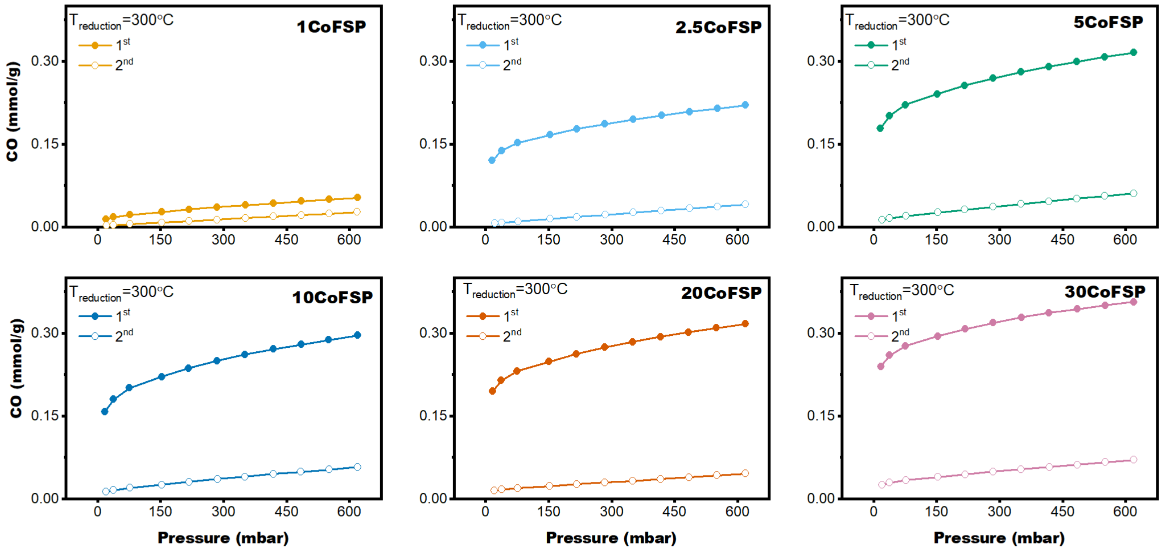

Figure S8. CO chemisorption at 35 °C results of CoFSP reduced at 300 °C for 4 h.

IR adsorption

Table S4. Assignment of IR bands.

Wavenumber (cm^{-1})	Assignment		Ref.
**2172; 2117**		rotovibrational spectrum of gas-phase CO	[8,9]
**2358**		CO_2 gas-phase	[10]
		**Ce**	
**2140-2125**	Ce^{3+}	CO ads	[11,12]
**2157**	Ce^{4+}	CO ads	
**1580 ν (CO), 1335 ν (CO), ~1370 δ (OCH)**	Formate I (on Ce^{3+})		[13–15]
**1561 ν (CO), 1356 ν (CO), ~1370 δ (OCH)**	Formate II (on Ce^{3+})		
**1550 ν (CO), 1371 ν (CO), ~1370 δ (OCH)**	Formate III (on Ce^{4+})		
**1400-1440; 1580-1585**		Monodentate carbonate (CO_3)	[16,17]
**1560-1567, 1289-1300, 1014 – 1030;**		Bidentate carbonate (CO_3)	[11,15,18]
**1490, 1380, 1085**	Ce^{3+}	Tridentate carbonate (CO_3)	[14]
**1451-1500; 1342-1380; 1038-1065**	Ce^{4+}	Tridentate carbonate (CO_3)	
**1465-1460; 1359-1460; 1080**		Polydentate/ monodentate carbonates (CO_3)	[11,12,16,18]
**1220-1225; 1420-1425; 1635-1640**		Bicarbonates (HCO_3)	[17]
		**Co**	
**2136**	Co_3O_4		[19]
**2125**	CoO		
**2120**	Co2+	CO adsorption	[20]
**2070**	Co2+	CO adsorption	
**2143-2180**	Co3+	CO adsorption	
**2015**	Co	a-top CO	[21,22]
**2000-2057**	Co	a-top CO on Co nanoparticles	
**2030-2040**	Co	a-top CO on Co clusters	
**1850-1862**	Co	hollow CO	[23,24]
**1930-1970**	Co	bridge CO	

IR-CO at 50 °C

Note S3.

The narrow carbonyl band in the 2020 – 2040 cm^{-1} range for 2.5CoFSP points to CO adsorption on very small Co clusters [25–27]. It is unlikely that atomically reduced Co is stable on CeO_2. In line with this, the spectra of 2.5CoFSP also contain a band due to bridged carbonyls. With increasing Co content, the position of the linear carbonyl band shifts to lower wavenumbers (2000 cm^{-1}. This carbonyl band is already significantly broader at low CO coverage compared to the carbonyl band in 2.5CoFSP. This can be explained by CO adsorption on larger metallic Co particles in reduced 5CoFSP, 10CoFSP and 20CoFSP. With increasing CO partial pressure, the carbonyl band shifts to 2040 cm^{-1} due to lateral interactions between adsorbed CO molecules, which is typical for metallic Co nanoparticles [10,22]. The IR spectra of the CoFSP samples also contain indications of various formate species (formate-I at 2838, 1584, and 1565 cm^{-1}; formate-II at 2838, 1565, and 1359 cm^{-1}; formate-III at 1550, 1371 cm^{-1}) with a minor contribution of carbonate species (**Figure S9**) [28].

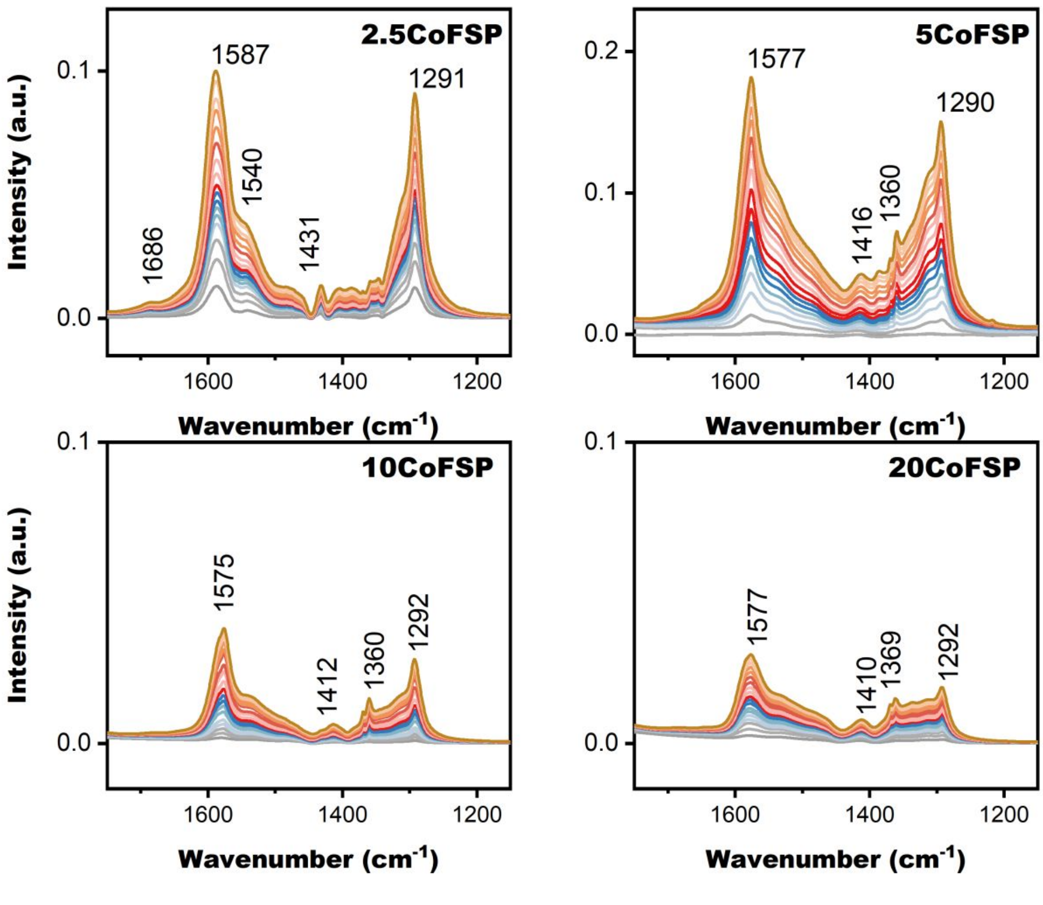

Figure S9. Carbonate region of the IR spectra of CoFSP catalysts reduced at 300 °C after CO adsorption at 50 °C (conditions: 0 – 10 mbar CO).

IR-CO at Liquid N_2 temperature

Note S4.

The corresponding IR spectra recorded at liquid N_2 temperature are shown in **Figure S10-11**. The carbonyl band for 2.5CoFSP is narrower than the carbonyl bands for the other samples, further supporting the conclusion that this sample contains small Co clusters. The relatively narrow band at ~1933 cm^{-1}, due to bridged carbonyls on Co clusters, was only observed for the reduced 2.5CoFSP. Moreover, the carbonyl bands on the small Co clusters in 2.5CoFSP are much broader in the IR spectra recorded at liquid N_2 temperature than those recorded at 50 °C. This is likely due to lateral interactions of these carbonyls with CO adsorbed on proximate Co^{2+} sites and other sites. While the reduced 2.5CoFSP sample likely contains very small metallic Co clusters, the reduced 5CoFSP, 10CoFSP, and 20CoFSP contain metallic Co nanoparticles, as evident from the IR spectra. With increasing Co content, the band at ~2030 cm^{-1} broadens and shifts to ~2055 cm^{-1}.

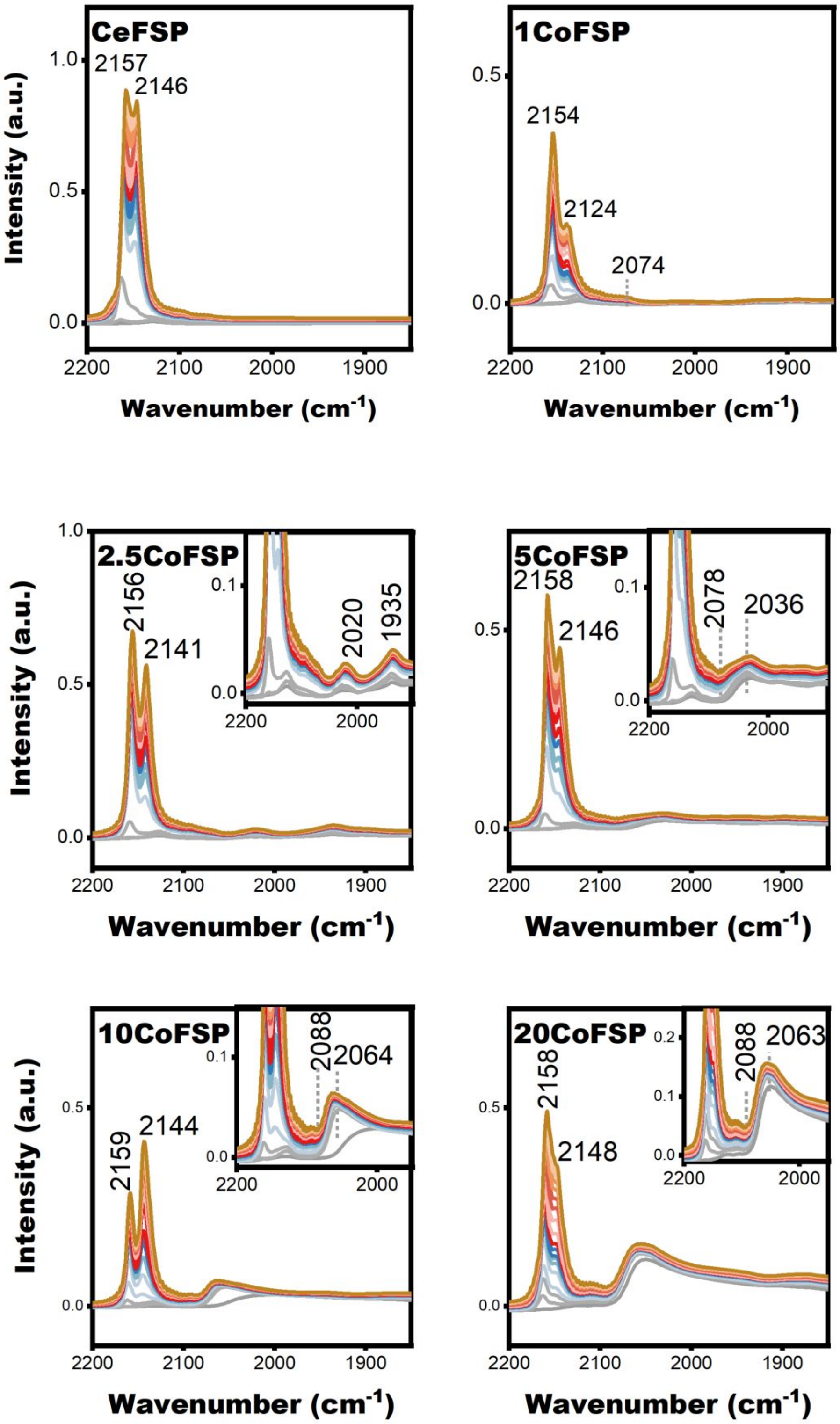

Figure S10. IR spectra of the CeFSP and CoFSP reduced at 300 °C after CO adsorption at liquid N_2 temperature (conditions: 0 (grey) – 10 (orange) mbar CO).

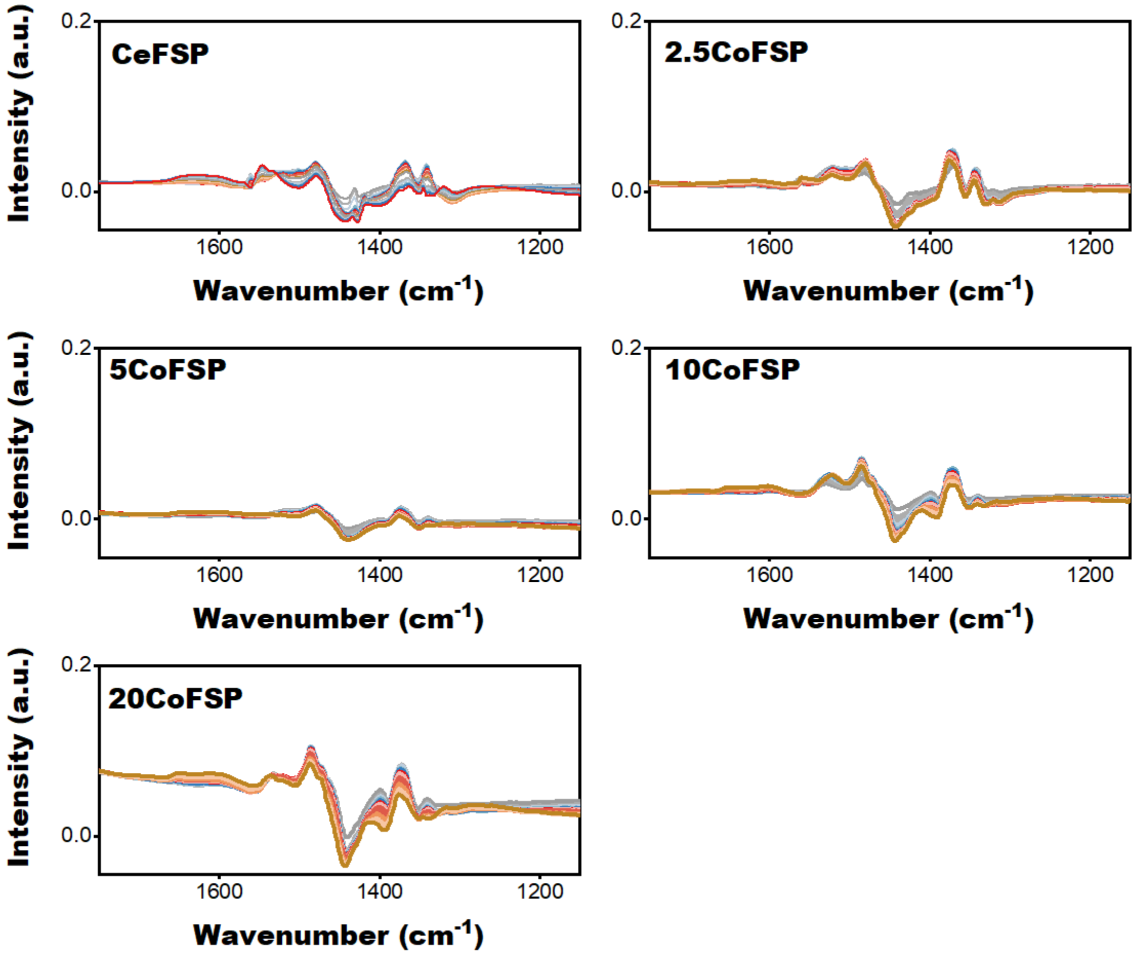

Figure S11. Carbonate regions of IR spectra of the CeFSP and CoFSP reduced at 300 °C after CO adsorption at liquid N_2 temperature (conditions: 0 – 10 mbar CO).

IR-CO_2

Note S5.

All IR spectra contain a strong band due to the asymmetric stretching vibration of gaseous CO_2 at ~2346 cm^{-1} [10]. Notably, the carbonyl bands for 2.5CoFSP are narrower and sharper than those for the other samples, which aligns with the CO IR spectra presented above(**Figure S12)**. At a Co content of 5 mol.% and above, the spectra contain a strong carbonyl feature at 2000 cm^{-1} due to CO adsorbed on Co nanoparticles. Compared to 2.5CoFSP, the IR spectra of 2.5CoFSP, the 5CoFSP, 10CoFSP, and 20CoFSP catalysts contained much stronger carbonyl bands. Notably, the (bi)carbonate/formate bands are stronger than the carbonyl bands for all CoFSP catalysts. Such formate and carbonate species might also be involved in CO2 dissociation.

The negative band at 2090 cm^{-1} in the CO_2 IR spectra indicates that CeO_2 was partially reoxidized by CO_2 in the reduced CoFSP catalysts. Moreover, a broad band was observed in the 2110 – 2135 cm^{-1} range for the reduced CeO_2-based catalysts, which is due to the $^2F5/2 \rightarrow {}^2F7/2$ electronic transition of Ce^{3+} [11]. The appearance of this band upon reduction evidences the partial reduction of CeO_2 in the CeFSP and CoFSP catalysts [29]. Various formate (formate-I at 2838, 1584, and 1565 cm^{-1}; formate-II at 2838, 1565 and 1359 cm^{-1}; formate-III at 1550, 1371 cm^{-1}) and carbonate and bicarbonate (1635, 1425, 1222 cm^{-1}) species are also observed for all CoFSP catalysts (**Figure S13**), which implies the high surface reactivity towards CO_2. Earlier, Parastaev et al. concluded that CO_2 activation in partially reduced Co/CeO_2-ZrO_2 catalysts takes place at sites at the interface between metallic Co and reducible components in the catalysts, namely CoO and CeO_2.[26]

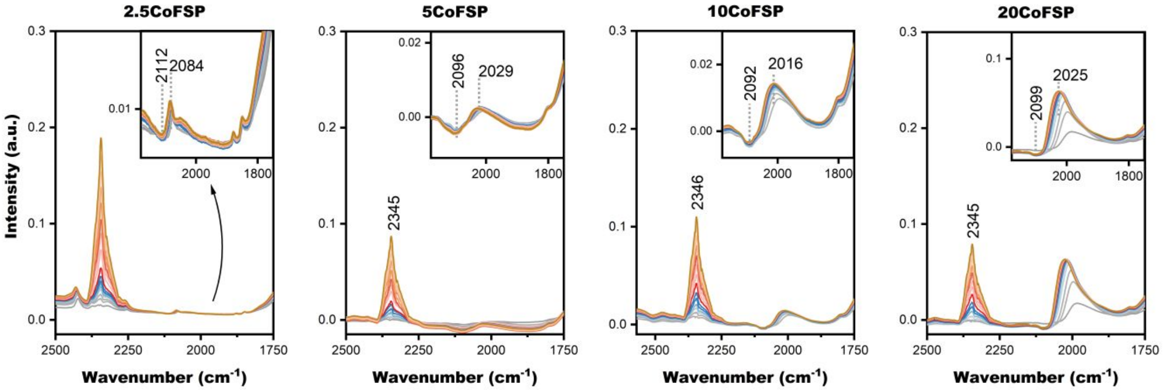

Figure S12. IR spectra of the CoFSP reduced at 300 °C after CO_2 adsorption at 50 °C (conditions: 1 – 10 mbar CO_2).

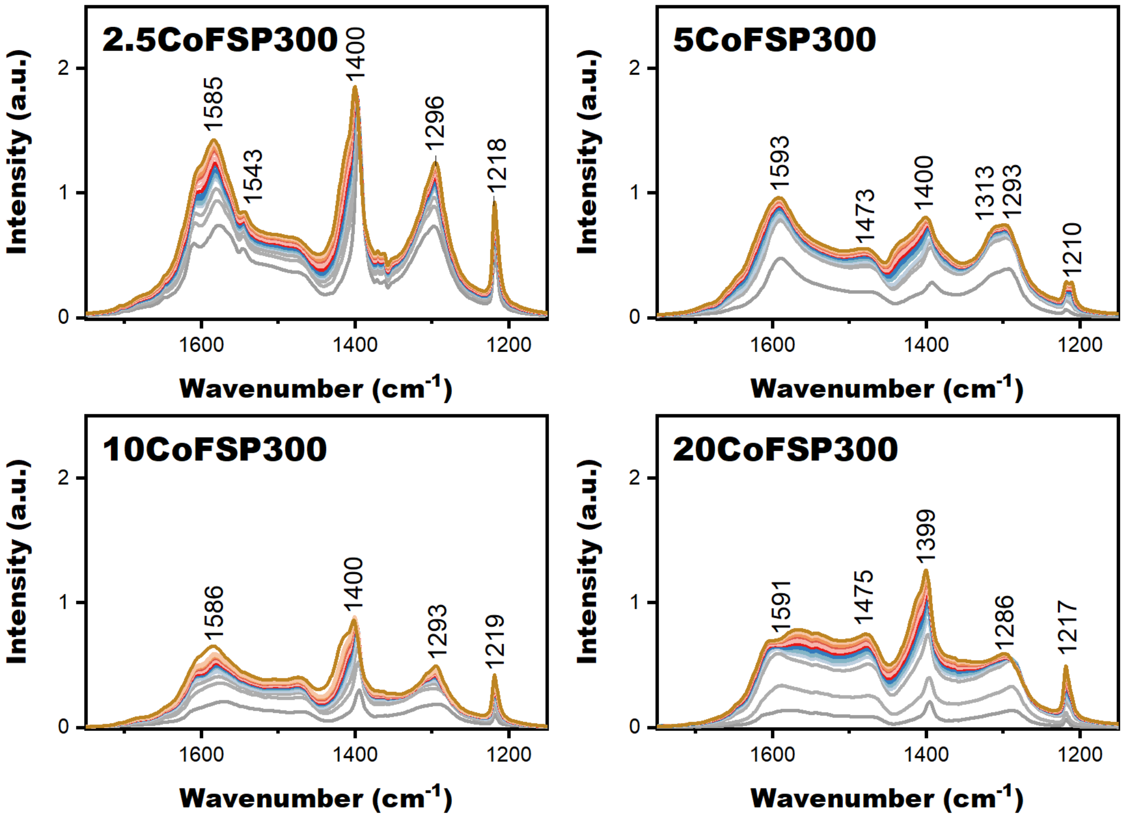

Figure S13. Carbonate region of IR spectra of CoFSP reduced at 300 °C after CO_2 adsorption at 50 °C (conditions: 0 – 10 mbar CO_2).

Structural changes of CeFSP and CoFSP during reduction (XRD and PDF)

Note S6.

The structural changes of the CoFSP catalysts and the bare CeFSP support during reduction and subsequent CO_2 hydrogenation were also investigated by in situ synchrotron XRD. The XRD patterns of all CoFSP catalysts can be described well by CeO_2 (**Figure S14-15**). No reflections related to Co-containing phases were observed on 2.5CoFSP and 5CoFSP, suggesting that the reduced Co particles were very small, which is in line with the STEM-EDX maps. The 10CoFSP and 20CoFSP show a stepwise reduction of Co_3O_4 to Co metal following the sequence $Co_3O_4 \rightarrow CoO \rightarrow Co$ at temperatures around 175 °C and 225 °C, respectively (**Figure S14-15**). The ΔXRD shows that, with increasing Co content, the contribution at q = 2.96 $Å^{-1}$ due to metallic Co becomes stronger (**Figure S15a**). The negative features at q = 2.58 and 4.36 $Å^{-1}$ in the subtracted XRD patterns (ΔXRD) of the 10CoFSP and 20CoFSP catalysts are due to the reduction of Co_3O_4.

Rietveld refinement of the XRD patterns revealed an increase in the CeO_2 unit cell parameter during reduction for all catalysts and the CeFSP sample (**Figure S15b**), along with a minor increase in the CeO_2 crystallite size from 8 nm to 12 nm (**Figure S15c**). In addition to gradual thermal expansion, a more abrupt CeO_2 unit cell parameter expansion from 5.405 to 5.444 Å was observed in the 100 – 250 °C temperature range for all CoFSP catalysts and the CeFSP support (**Figure S15b**). Nevertheless, the expansion of the CeFSP unit cell is more gradual, and the change in the unit cell parameter is less pronounced than that of the CoFSP catalysts (**Figure S15b**). The reduction of Ce^{4+} to the larger Ce^{3+} ion and the electrostatic repulsion between oxygen vacancies and the surrounding cations cause this abrupt increase of the unit cell parameter [30].

The G(r) derived from the PDF of CoFSP and CeFSP before and after reduction are shown in **Figure S16**. The observed peaks at distances r = 3.8, 4.5, 5.4, 5.9, and 6.6 Å are characteristic of the CeO_2 structure. The refined lattice parameters of as-prepared CeFSP, 2.5CoFSP, 5CoFSP, and 10CoFSP catalysts are 5.408 Å, 5.398 Å, 5.399 Å, and 5.400 Å, respectively (**Table S5**). The decrease in the unit cell parameter for CoFSP samples can be explained by the shortening of the average distance between Ce-Ce atom pairs due to Co insertion in the CeO_2 lattice, which is in good agreement with the other characterization results [31]. The change in the unit cell parameter is less pronounced for the catalysts containing more Co. The experimental PDF curves were also fitted by a Gaussian to the expected peak at a Ce-Ce distance of ~3.8 Å in the CeO_2 fluorite structure. The resulting CeO_2 peaks of as-prepared CoFSP catalysts exhibit shorter interatomic distances than the CeFSP peaks, which supports the insertion of Co in the CeO_2 structure (**Table S6**). To identify small changes due to Co, ΔG(r) was determined by subtracting G(r) of CeFSP from the G(r) of the CoFSP catalysts (**Figure S17**). Following an example in literature for Ni-ZrO_2-CeO_2 [32], the PDFs of Co_3O_4, CoO, Co, and Co doped in CeO_2 were modeled. Comparison of the experimental PDF to these models shows that the ΔG(r) of the as-prepared 10CoFSP catalyst contains features of Co_3O_4 and Co doped in the CeO_2 lattice (r = 3.8 and 4.5 Å) (**Figure S17**), while the ΔG(r) of the 2.5CoFSP and 5CoFSP shows mostly peaks matching those of Co doped in the CeO_2 lattice (r = 3.8 and 4.5 Å).

The direct G(r) analysis and Rietveld refinement of the PDF data of the reduced catalysts show an increase in the interatomic distances, implying an expansion of the CeO_2 lattice. This can be attributed to thermal expansion and the formation of Ce^{3+} ions. The likely random distribution of Ce^{3+} in the reduced catalysts and the increased thermal motion at higher temperatures can explain the broader peaks in the PDF (**Figure S15d**). The ΔG(r) of the reduced CoFSP catalysts shows features of metallic Co (r

= 2.5; 4.3 and 5.6 Å) and Co doped into the CeO_2 lattice (r = 3.8 and 4.5 Å) (**Figure S17**). The latter implies that not all Co^{2+} ions were reduced under these conditions, which is consistent with the quasi-in situ XPS and STEM-EDX results of the reduced catalysts.

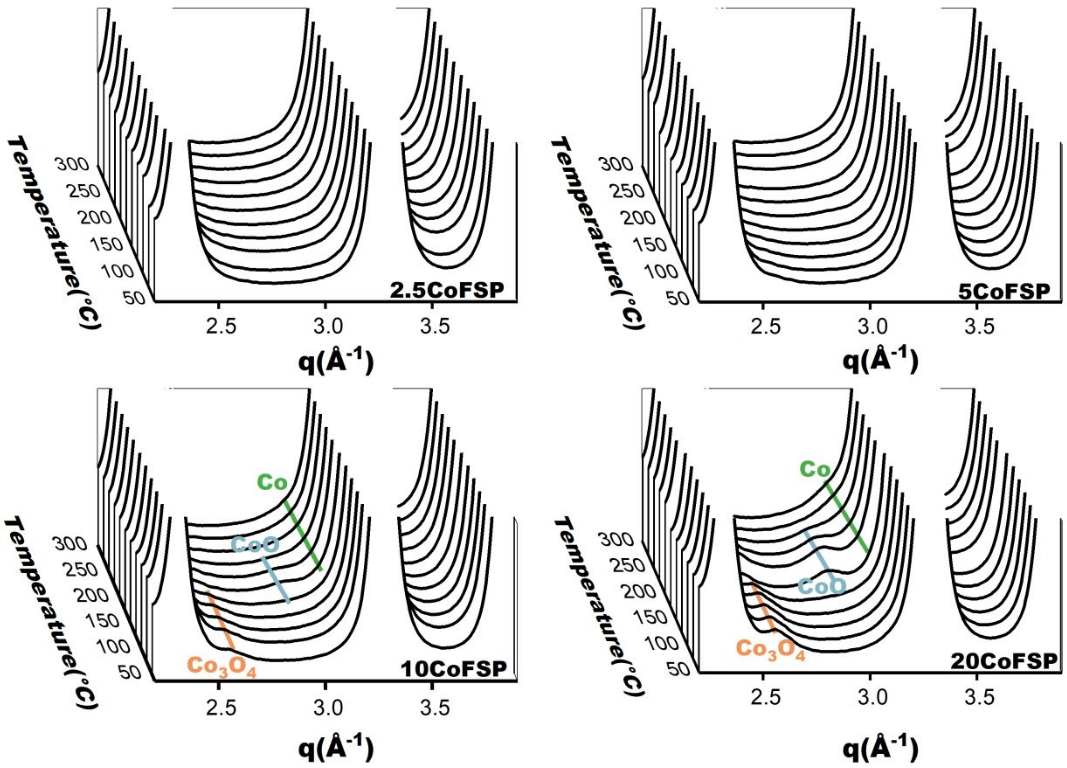

Figure S14. Synchrotron XRD diffractograms of CoFSP during reduction (conditions: 20 vol.% H_2 in Ar, 50 mL/min, 1 bar, 50 – 300 °C, 8.5 °C/min).

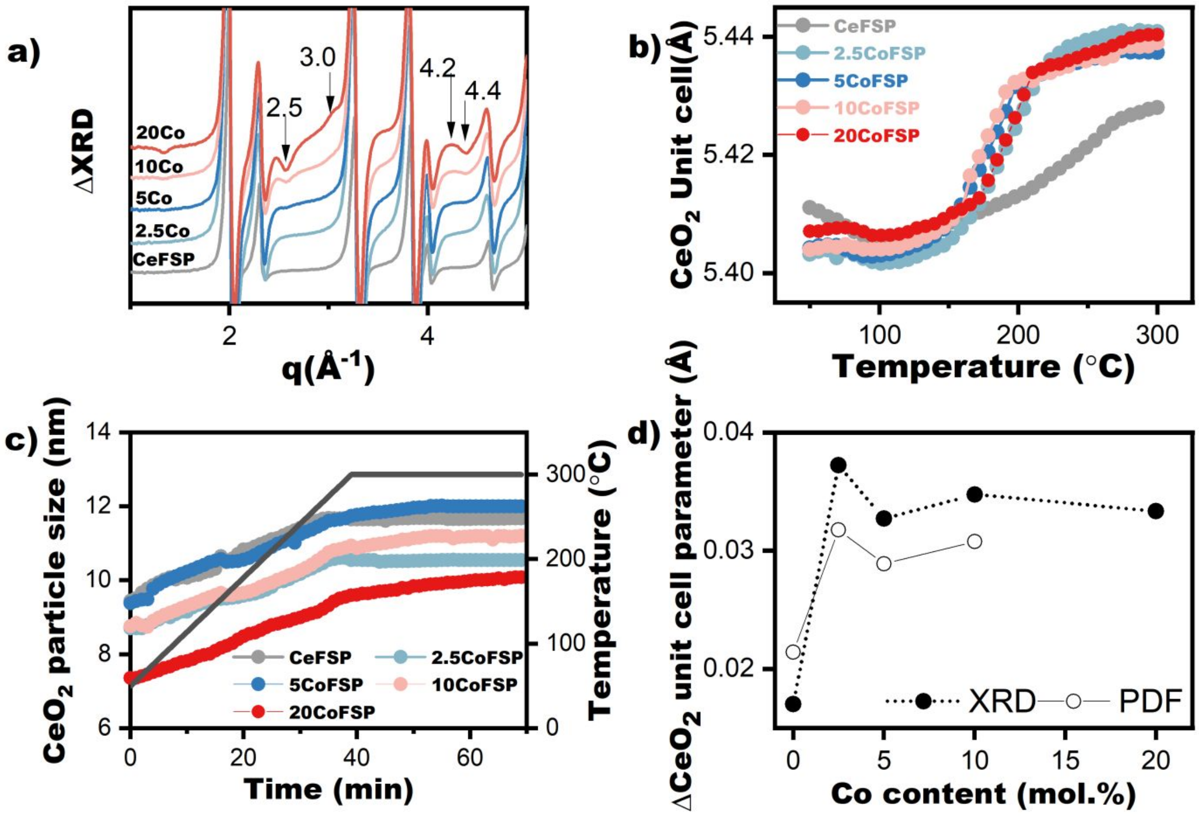

Figure S15. (a) Synchrotron ΔXRD (λ = 0.124 Å) of CeFSP and CoFSP before and after reductive pretreatment. ΔXRD difference is obtained by subtraction of the diffractogram of as-prepared sample from reduced diffractogram of the same sample. (b) Refined unit cell parameter of CeO_2 for CeFSP and CoFSP during reductive pretreatment. (c) Refined CeO_2 particle size of CeFSP and CoFSP during pretreatment (conditions: 20 vol.% H_2 in Ar, 50 mL/min, 50 – 300 °C, 1 bar). (d) ΔCeO_2 unit cell parameter of CeFSP and CoWI, derived from XRD and PDF results. ΔCeO_2 unit cell parameter was obtained by substruction of CeO_2 unit cell parameter of as-prepared sample from CeO_2 unit cell parameter of the same sample after reduction.

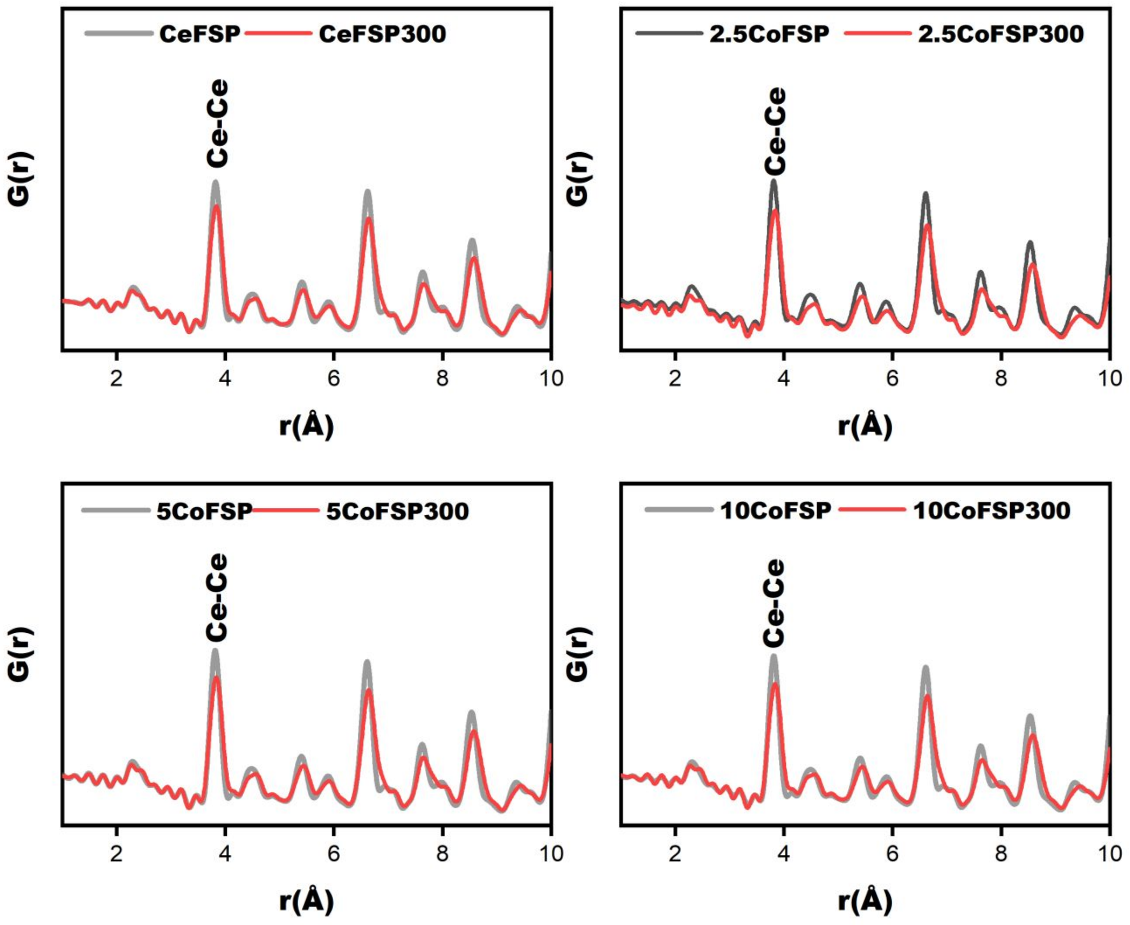

Figure S16. G(r) of CeFSP and CoFSP before and after reduction (conditions: 20 vol.% H_2 in Ar, 50 mL/min, 1 bar, 50 – 300 °C, 8.5 °C/min).

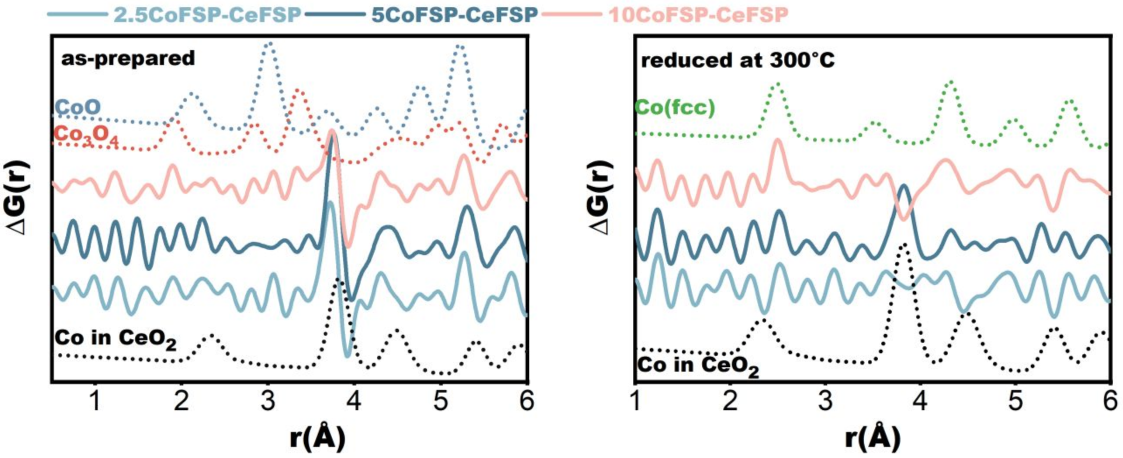

Figure S17. ΔG(r) of CoFSP and modeled G(r) of Co_3O_4, CoO, Co metal, and Co incorporated into CeO_2. ΔG(r) is obtained by subtraction of G(r) of CeFSP from G(r) of CoFSP recorded under the same conditions (pretreatment conditions: 20 vol.% H_2 in Ar, 50 mL/min, 1 bar, 50 – 300 °C, 8.5 °C/min).

Table S5. Results of Rietveld refinement of PDF of CeFSP and CoFSP using CeO_2 fluorite model (error margins are reported in brackets).

Sample	a (Å) (error)	dCeO2 (Å)	Rw
**CeFSP**	5.40807 (0.00058)	59.9 (2.7)	0.13811
**CeFSP300**	5.42949 (0.00082)	67.6 (4.4)	0.147708
**2.5CoFSP**	5.39847 (0.00064)	52.4 (2.0)	0.147665
**2.5CoFSP300**	5.43021 (0.00093)	57.7 (3.1)	0.150841
**5CoFSP**	5.39869 (0.00056)	58.5 (2.4)	0.146947
**5CoFSP300**	5.42758 (0.0008)	67.1 (4.1)	0.142615
**10CoFSP**	5.40019 (0.00061)	55.1 (2.2)	0.145461
**10CoFSP300**	5.43095 (0.0009)	62.5 (3.8)	0.147448

Table S6. Results of direct PDF analysis of CeFSP and CoFSP.

Sample	FWHM (A) Gauss function	Position	Area (a.u.)
**CeFSP**	0.113	3.821	2.62665
**CeFSP300**	0.145	3.838	2.73816
**2.5CoFSP**	0.113	3.813	2.65109
**2.5CoFSP300**	0.145	3.838	2.79109
**5CoFSP**	0.110	3.814	2.71007
**5CoFSP300**	0.145	3.837	2.83795
**10CoFSP**	0.111	3.815	2.60705
**10CoFSP300**	0.149	3.837	2.72874

Comparison of CO_2 hydrogenation performance over different catalysts

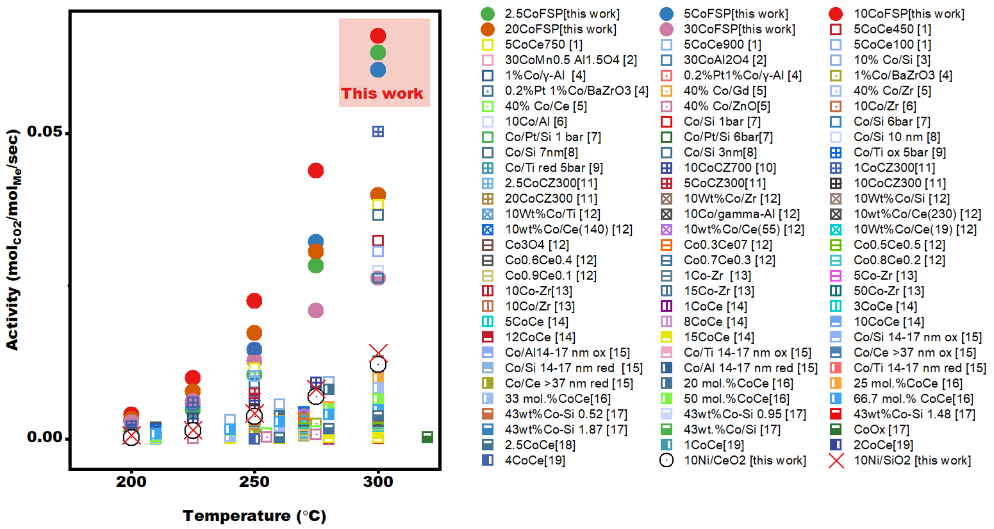

Figure S18. Catalytic activity of CoFSP in comparison to literature.

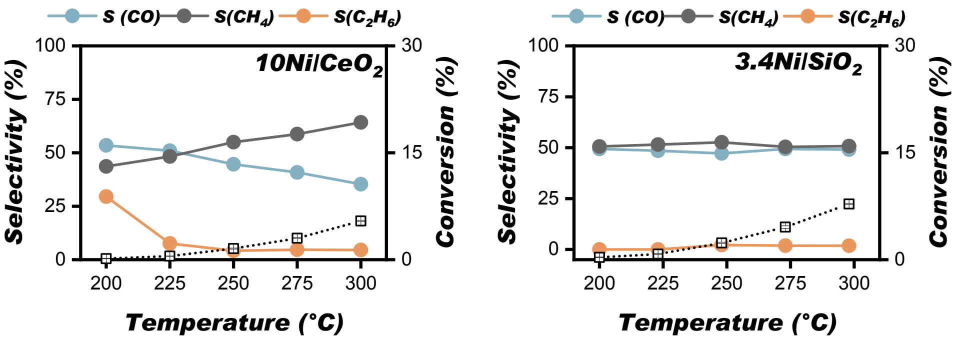

Figure S19. Catalytic performance of 10Ni/CeO_2 (left) and Ni/SiO_2 (right) catalysts reduced at 300 °C in CO_2 hydrogenation as a function of temperature (conditions: 200 – 300 °C, 50 mg of catalyst, 15 vol.% CO_2, 60 vol.% H_2, 25 vol.% Ar, 50 mL/min, 1 bar).

Table S7. Catalytic performance in CO_2 hydrogenation.

Catalyst	H_2: CO_2	P (bar)	CO_2 initial (%)	T_{react} (°C)	Activity*10^{-3} ($molCO_2$/molMe/s)	Ref. Fig. S17	Ref.
**2.5CoFSP**	4	1	15	200	**1.23**	This work	This work
				225	**4.72**		
				250	**10.43**		
				275	**28.30**		
				300	**63.22**		
**5CoFSP**				200	**3.04**		
				225	**6.23**		
				250	**18.97**		
				275	**34.90**		
				300	**52.53**		
**10CoFSP**				200	**3.92**		
				225	**9.89**		
				250	**22.37**		
				275	**43.49**		
				300	**65.38**		
**20CoFSP**				200	**4.52**		
				225	**10.75**		
				250	**24.08**		
				275	**42.59**		
				300	**55.52**		
**30CoFSP**				200	**4.35**		
				225	**9.90**		
				250	**20.59**		
				275	**33.97**		
				300	**42.53**		
**10Ni/CeO_2**				200	**0.14**		
				225	**1.32**		
				250	**3.66**		
				275	**6.85**		
				300	**12.11**		
**10Ni/SiO_2**				200	**0.58**		
				225	**1.42**		
				250	**4.19**		
				275	**8.18**		
				300	**13.98**		

Table S7. Catalytic performance in CO_2 hydrogenation (continuation).

Catalyst	H_2: CO_2	P (bar)	CO_2 initial (%)	T_{react} (°C)	Activity*10^{-3} (molCO_2/molMe/s)	Ref. Fig. S18	Ref.
**5Co/Ce450**	1	1	45	250	**10.31**	[1]	33
				300	**32.49**		
**5Co/Ce750**				250	**11.50**		
				300	**38.36**		
**5Co/Ce900**				250	**9.21**		
				300	**30.68**		
**5Co/Ce1000**				250	**7.66**		
				300	**30.68**		
**30Co$Mn_{0.5}Al_{1.5}O_4$**	4	1		200	**0.74**	[2]	34
				250	**4.56**		
				300	**9.73**		
				350	**10.50**		
				400	**10.70**		
				450	**10.60**		
				500	**10.30**		
**30CoAl**				300	**0.25**		
				350	**1.78**		
				400	**6.22**		
				450	**8.28**		
				500	**8.51**		
**10% Co/Si**	4	1	10	240	**3.11**	[3]	35
				260	**5.60**		
				280	**9.35**		
				300	**12.70**		
				320	**15.70**		
				340	**17.80**		
				360	**19.40**		
				380	**21.10**		
				400	**22.10**		
**1%Co/γ-Al**	4	1	3	300	**0.91**	[4]	36
				325	**2.40**		
				350	**4.05**		
**0.2%Pt1%Co/γ-Al**				300	**2.02**		
				325	**4.97**		
				350	**7.90**		
**1%Co/$BaZrO_3$**				275	**2.52**		
				300	**6.01**		
				325	**10.50**		
				350	**13.70**		
**0.2%Pt 1%Co/$BaZrO_3$**				250	**3.06**		
				275	**7.33**		
				300	**11.80**		
				325	**16.90**		

Si – SiO_2; Ce – CeO_2; CZ – CeO_2-ZrO_2; Al – Al_2O_3; Zr – ZrO_2

Table S7. Catalytic performance in CO_2 hydrogenation (continuation).

Catalyst	H_2: CO_2	P (bar)	CO_2 initial (%)	T_{react} (°C)	Activity*10^{-3} (molCO_2/molMe/s)	Ref. Fig. S18	Ref.
**40% Co/Gd**	9	1	10	210	**0.49**	[5]	37
				225	**0.63**		
				240	**0.76**		
				255	**0.85**		
				270	**0.90**		
				285	**0.91**		
				300	**0.92**		
**40% Co/Zr**				210	**0.34**		
				225	**0.48**		
				240	**0.54**		
				255	**0.54**		
				270	**0.56**		
				285	**0.60**		
				300	**0.70**		
**40% Co/Ce**				210	**0.31**		
				225	**0.46**		
				240	**0.70**		
				255	**0.90**		
				270	**1.06**		
				285	**1.14**		
				300	**1.19**		
**40% Co/ZnO**				210	**0.06**		
				225	**0.11**		
				240	**0.18**		
				255	**0.25**		
				270	**0.34**		
				285	**0.58**		
				300	**0.74**		
**10% Co/Zr**	4	30	20	400	**12.00**	[6]	37
**10% Co/Al**				400	**16.00**		
**Co/Si**	4	1	22.2	200	**0.08**	[7]	38
		6		200	**0.25**		
**Co/Pt/Si**		1		200	**0.46**		
		6		200	**1.08**		
**Co/Si 10nm**	4	6	22.2	200	**0.59**	[8]	39
				250	**10.10**		
				300	**27.50**		
**Co/Si 7 nm**				200	**1.54**		
				250	**8.99**		
				300	**26.30**		
**Co/Si 3nm**				200	**1.40**		
				250	**6.48**		
				300	**36.60**		
**Co/Ti (ox 5bar)**	4	5	25	250	**3.20**	[9]	40
**Co/Si (red 5bar)**				250	**1.30**		

Ce – CeO_2; Zr – ZrO_2; Si – SiO_2; Al – Al_2O_3; Gd -Gd_2O_3

Table S7. Catalytic performance in CO_2 hydrogenation (continuation).

Catalyst	H_2: CO_2	P (bar)	CO_2 initial (%)	T_{react} (°C)	Activity*10^{-3} ($molCO_2/molMe/s$)	Ref. Fig. S18	Ref.
**10CoCZ700 (red 500 °C)**	4	1	5	200	**1.46**	[10]	29
				225	**3.35**		
				250	**6.22**		
				275	**9.19**		
				300	**11.30**		
				325	**12.80**		
**1CoCZ300**	4	1	5	250	**14.65**	[11]	26
**2.5CoCZ300**				250	**10.23**		
**5CoCZ300**				250	**7.37**		
**10CoCZ300**				250	**4.78**		
**20CoCZ300**				250	**2.53**		
**10wt%Co/Ce (55)**	4	1	10	200	**0.31**	[12]	41
				220	**0.69**		
				250	**2.36**		
				270	**3.88**		
				300	**4.30**		
**10Wt%Co/Ce(19)**				200	**0.23**		
				220	**0.41**		
				250	**1.42**		
				270	**3.23**		
				300	**4.22**		
**Co_3O_4**				200	**0.07**		
				220	**0.18**		
				250	**0.38**		
				270	**0.44**		
				300	**0.44**		
**$Co_{0.3}Ce_{0.7}$**				200	**0.03**		
				220	**0.40**		
				250	**2.37**		
				270	**3.29**		
				300	**3.41**		
**$Co_{0.5}Ce_{0.5}$**				200	**0.04**		
				220	**0.29**		
				250	**1.26**		
				270	**1.70**		
				300	**1.72**		
**$Co_{0.6}Ce_{0.4}$**				200	**0.21**		
				220	**0.49**		
				250	**1.08**		
				270	**1.28**		
				300	**1.29**		

Ti – TiO_2; Si – SiO_2; Ce – CeO_2; CZ – CeO_2-ZrO_2.

Table S7. Catalytic performance in CO_2 hydrogenation (continuation).

Catalyst	H_2: CO_2	P (bar)	CO_2 Initial (%)	T_{react} (°C)	Activity*10^{-3} (molCO_2/molMe/s)	Ref. Fig. S18	Ref.
**$Co_{0.7}Ce_{0.3}$**				200	**0.25**		
				220	**0.54**		
				250	**0.99**		
				270	**1.02**		
				300	**1.02**		
**$Co_{0.8}Ce_{0.2}$**				200	**0.19**		
				220	**0.63**		
				250	**1.02**		
				270	**1.02**		
				300	**1.02**		
**Co0.9Ce0.1**	4	1	10	200	**0.27**		
				220	**0.50**		
				250	**0.58**		
				270	**0.58**		
				300	**0.58**		
**10wt%Co/Zr**	50	1	1	200	**0.62**	[12]	41
				220	**1.24**		
				250	**2.60**		
				270	**3.77**		
				300	**4.27**		
**10wt%Co/Si**				200	**0.42**		
				220	**1.24**		
				250	**3.26**		
				270	**4.07**		
				300	**4.38**		
**10wt%Co/Ti**				200	**0.05**		
				220	**0.11**		
				250	**0.39**		
				270	**0.92**		
				300	**1.39**		
**10Co/gamma-Al**				200	**0.06**		
				220	**0.24**		
				250	**0.64**		
				270	**1.47**		
				300	**2.16**		
**10wt%Co/Ce (230)**				200	**0.91**		
				220	**1.81**		
				250	**3.63**		
				270	**4.41**		
				300	**4.38**		

Si – SiO_2; Ce – CeO_2; CZ – CeO_2-ZrO_2; Al – Al_2O_3; Zr – ZrO_2

Table S7. Catalytic performance in CO_2 hydrogenation (continuation).

Catalyst	H_2: CO_2	P (bar)	CO_2 Initial (%)	T_{react} (°C)	Activity*10^{-3} (molCO_2/molMe/s)	Ref. Fig. S18	Ref.
**10wt%Co/Ce (140)**				200	**0.72**	[12]	41
				220	**1.58**		
				250	**3.57**		
				270	**4.39**		
				300	**4.39**		
**1Co-Zr**	4	30	0.1667	340	**0.02**	[13]	42
**5Co-Zr**				340	**0.08**		
**10Co-Zr**				340	**0.10**		
**15Co-Zr**				340	**0.10**		
**50Co-Zr**				340	**0.40**		
**10Co/Zr**				340	**0.45**		
**10Co-Zr**				280	**0.01**		
**10Co-Zr**				300	**0.01**		
**1wt%Co/Ce**	4	1	0.1	220	**0.11**	[14]	43
				240	**0.11**		
				260	**0.11**		
				280	**0.11**		
				300	**0.33**		
**3wt%Co/Ce**				220	**0.04**		
				240	**0.04**		
				260	**0.11**		
				280	**0.26**		
				300	**0.33**		
**5wt%Co/Ce**				220	**0.00**		
				240	**0.02**		
				260	**0.09**		
				280	**0.18**		
				300	**0.24**		
**8wt%Co/Ce**				220	**0.03**		
				240	**0.05**		
				260	**0.10**		
				280	**0.16**		
				300	**0.21**		
**10wt%Co/Ce**				220	**0.02**		
				240	**0.05**		
				260	**0.10**		
				280	**0.14**		
				300	**0.18**		

Si – SiO_2; Ce – CeO_2; CZ – CeO_2-ZrO_2; Al – Al_2O_3; Zr – ZrO_2

Table S7. Catalytic performance in CO_2 hydrogenation (continuation).

**12wt%Co/Ce**	**4**	**1**	**0.1**	**220**	**0.03**	**[14]**	43
				240	**0.06**		
				260	**0.10**		
				280	**0.14**		
				300	**0.16**		
**15wt%Co/Ce**				220	**0.03**		
				240	**0.06**		
				260	**0.10**		
				280	**0.12**		
				300	**0.13**		
**Co/Si [14-17 nm] ox**	3	20	24	250	**0.22**	[15]	44
**Co/Al [14-17 nm] ox**				250	**0.23**		
**Co/Ti [14-17 nm] ox**				250	**0.95**		
**Co/Ce [>37 nm] ox**				250	**0.57**		
**Co/Si [14-17 nm] red**				250	**0.58**		
**Co/Al [14-17 nm] red**				250	**0.27**		
**Co/Ti [14-17 nm] red**				250	**0.75**		
**Co/Ce [>37 nm] red**				250	**0.70**		
**20mol%Co/Ce**	4	1	10	220	**0.84**	[16]	45
				240	**1.53**		
				260	**3.73**		
				280	**8.03**		
				300	**12.66**		
**25mol%Co/Ce**				220	**0.33**		
				240	**1.51**		
				260	**2.88**		
				280	**5.23**		
				300	**9.98**		
**33mol.%Co/Ce**				220	**0.14**		
				240	**0.38**		
				260	**2.04**		
				280	**4.81**		
				300	**8.27**		

Ti – TiO_2; Si – SiO_2; Ce – CeO_2; CZ – CeO_2-ZrO_2; Al - Al_2O_3

Table S7. Catalytic performance in CO_2 hydrogenation (continuation).

**50mol.%Co/Ce**	**4**	**1**	**10**	**220**	**0.42**		
				240	**1.43**		
				260	**2.82**		
				280	**4.79**		
				300	**6.51**		
**66.7mol%Co/Ce**				220	**0.81**		
				240	**1.58**		
				260	**2.77**		
				280	**3.90**		
				300	**4.70**		
**43wt%Co-Si 0.52**	2	20	25	320	**0.22**	[17]	46
**43wt%Co-Si 0.95**				320	**0.21**		
**43wt%Co-Si 1.48**				320	**0.20**		
**43wt%Co-Si 1.87**				320	**0.17**		
**43wt.%Co/Si**				320	**0.18**		
**CoOx**				320	**0.31**		
**2.5CoCe (Ce/Co=4)**	4	1	1	260	**0.01**	[18]	47
				280	**0.07**		
				300	**0.16**		
**1CoCe**	5	1	14.28	250	**0.0013**	[19]	48
**2CoCe**				250	**0.0012**		
**4CoCe**				250	**0.0007**		

.

References

(1) Zhang, S.; Xia, Z.; Zhang, M.; Zou, Y.; Shen, H.; Li, J.; Chen, X.; Qu, Y. Boosting Selective Hydrogenation through Hydrogen Spillover on Supported-Metal Catalysts at Room Temperature. Appl Catal B 2021, 297, 120418. https://doi.org/10.1016/J.APCATB.2021.120418.

(2) Ghogia, A. C.; Cayez, S.; Machado, B. F.; Nzihou, A.; Serp, P.; Soulantica, K.; Pham Minh, D. Hydrogen Spillover in the Fischer-Tropsch Synthesis on Carbon-Supported Cobalt Catalysts. ChemCatChem 2020, 12 (4), 1117–1128. https://doi.org/10.1002/CCTC.201901934.

(3) Chen, S.; Cao, T.; Gao, Y.; Li, D.; Xiong, F.; Huang, W. Probing Surface Structures of CeO_2, TiO_2, and Cu_2O Nanocrystals with CO and CO_2 Chemisorption. Journal of Physical Chemistry C 2016, 120 (38), 21472–21485. https://doi.org/10.1021/ACS.JPCC.6B06158.

(4) Yang, Z.; He, B.; Lu, Z.; Hermansson, K. Physisorbed, Chemisorbed, and Oxidized CO on Highly Active Cu-CeO_2(111). Journal of Physical Chemistry C 2010, 114 (10) , 4486–4494. https://doi.org/10.1021/JP909174U.

(5) Bridge, M. E.; Comrie, C. M.; Lambert, R. M. Hydrogen Chemisorption and the Carbon Monoxide-Hydrogen Interaction on Cobalt (0001). J Catal 1979, 58 (1), 28–33. https://doi.org/10.1016/0021-9517(79)90240-9.

(6) Weststrate, C. J.; van de Loosdrecht, J.; Niemantsverdriet, J. W. Spectroscopic Insights into Cobalt-Catalyzed Fischer-Tropsch Synthesis: A Review of the Carbon Monoxide Interaction with Single Crystalline Surfaces of Cobalt. J Catal 2016, 342, 1–16. https://doi.org/10.1016/J.JCAT.2016.07.010.

(7) Yang, J.; Frøseth, V.; Chen, D.; Holmen, A. Particle Size Effect for Cobalt Fischer–Tropsch Catalysts Based on in Situ CO Chemisorption. Surf Sci 2016, 648, 67–73. https://doi.org/10.1016/J.SUSC.2015.10.029.

(8) Meunier, F. C. On the Contamination with Nickel and Nickel Tetracarbonyl during FT-IR Investigation of Catalysts under CO-Containing Gases. J Catal 2019, 372, 388. https://doi.org/10.1016/J.JCAT.2019.03.006.

(9) Paredes-Nunez, A.; Jbir, I.; Bianchi, D.; Meunier, F. C. Spectrum Baseline Artefacts and Correction of Gas-Phase Species Signal during Diffuse Reflectance FT-IR Analyses of Catalysts at Variable Temperatures. Appl Catal A Gen 2015, 495, 17–22. https://doi.org/10.1016/J.APCATA.2015.01.042.

(10) Chen, W.; Zijlstra, B.; Filot, I. A. W.; Pestman, R.; Hensen, E. J. M. Mechanism of Carbon Monoxide Dissociation on a Cobalt Fischer–Tropsch Catalyst. ChemCatChem 2018, 10 (1), 136–140. https://doi.org/10.1002/CCTC.201701203.

(11) Binet, C.; Badri, A.; Boutonnet-Kizling, M.; Lavalley, J. C. FTIR Study of Carbon Monoxide Adsorption on Ceria: $CO^{2-}{}_2$ Carbonite Dianion Adsorbed Species. Journal of the Chemical Society, Faraday Transactions 1994, 90 (7), 1023–1028. https://doi.org/10.1039/FT9949001023.

(12) Binet, C.; Daturi, M.; Lavalley, J. C. IR Study of Polycrystalline Ceria Properties in Oxidised and Reduced States. Catal Today 1999, 50 (2), 207–225. https://doi.org/10.1016/S0920-5861(98) 00504-5.

(13) Lustemberg, P. G.; Bosco, M. V.; Bonivardi, A.; Busnengo, H. F.; Ganduglia-Pirovano, M. V. Insights into the Nature of Formate Species in the Decomposition and Reaction of Methanol over Cerium Oxide Surfaces: A Combined Infrared Spectroscopy and Density Functional Theory Study. Journal of Physical Chemistry C 2015, 119 (37), 21452–21464. https://doi.org/10.1021/ACS.JPCC.5B05070.

(14) Vayssilov, G. N.; Mihaylov, M.; Petkov, P. S.; Hadjiivanov, K. I.; Neyman, K. M. Reassignment of the Vibrational Spectra of Carbonates, Formates, and Related Surface Species on Ceria: A

Combined Density Functional and Infrared Spectroscopy Investigation. Journal of Physical Chemistry C 2011, 115 (47), 23435–23454. https://doi.org/10.1021/JP208050A.

(15) Li, C.; Sakata, Y.; Arai, T.; Domen, K.; Maruya, K. I.; Onishi, T. Adsorption of Carbon Monoxide and Carbon Dioxide on Cerium Oxide Studied by Fourier-Transform Infrared Spectroscopy. Part 2.—Formation of Formate Species on Partially Reduced CeO_2 at Room Temperature. Journal of the Chemical Society, Faraday Transactions 1: Physical Chemistry in Condensed Phases 1989, 85 (6), 1451–1461. https://doi.org/10.1039/F19898501451.

(16) Aguirre, A.; Collins, S. E. Selective Detection of Reaction Intermediates Using Concentration-Modulation Excitation DRIFT Spectroscopy. Catal Today 2013, 205, 34–40. https://doi.org/10.1016/J.CATTOD.2012.08.020.

(17) Vrijburg, W. L.; Moioli, E.; Chen, W.; Zhang, M.; Terlingen, B. J. P.; Zijlstra, B.; Filot, I. A. W.; Züttel, A.; Pidko, E. A.; Hensen, E. J. M. Efficient Base-Metal NiMn/TiO_2 Catalyst for CO_2 Methanation. ACS Catal 2019, 9 (9), 7823–7839. https://doi.org/10.1021/ACSCATAL.9B01968.

(18) Pozdnyakova, O.; Teschner, D.; Wootsch, A.; Kröhnert, J.; Steinhauer, B.; Sauer, H.; Toth, L.; Jentoft, F. C.; Knop-Gericke, A.; Paál, Z.; Schlögl, R. Preferential CO Oxidation in Hydrogen (PROX) on Ceria-Supported Catalysts, Part II: Oxidation States and Surface Species on Pd/CeO_2 under Reaction Conditions, Suggested Reaction Mechanism. J Catal 2006, 237 (1), 17–28. https://doi.org/10.1016/J.JCAT.2005.10.015.

(19) Mehl, S.; Ferstl, P.; Schuler, M.; Toghan, A.; Brummel, O.; Hammer, L.; Schneider, M. A.; Libuda, J. Thermal Evolution of Cobalt Deposits on Co_3O_4 (111): Atomically Dispersed Cobalt, Two-Dimensional CoO Islands, and Metallic Co Nanoparticles. Physical Chemistry Chemical Physics 2015, 17 (36), 23538–23546. https://doi.org/10.1039/C5CP03922C.

(20) Busca, G.; Guidetti, R.; Lorenzelli, V. Fourier-Transform Infrared Study of the Surface Properties of Cobalt Oxides. 1990, 86 (6), 989–994.

(21) Ferstl, P.; Mehl, S.; Arman, M. A.; Schuler, M.; Toghan, A.; Laszlo, B.; Lykhach, Y.; Brummel, O.; Lundgren, E.; Knudsen, J.; Hammer, L.; Schneider, M. A.; Libuda, J. Adsorption and Activation of CO on Co_3O_4(111) Thin Films. Journal of Physical Chemistry C 2015, 119 (29), 16688–16699. https://doi.org/10.1021/ACS.JPCC.5B04145.

(22) Zijlstra, B.; Broos, R. J. P.; Chen, W.; Oosterbeek, H.; Filot, I. A. W.; Hensen, E. J. M. Coverage Effects in CO Dissociation on Metallic Cobalt Nanoparticles. ACS Catal 2019, 9 (8), 7365–7372. https://doi.org/10.1021/ACSCATAL.9B01967..

(23) Weststrate, C. J.; van de Loosdrecht, J.; Niemantsverdriet, J. W. Spectroscopic Insights into Cobalt-Catalyzed Fischer-Tropsch Synthesis: A Review of the Carbon Monoxide Interaction with Single Crystalline Surfaces of Cobalt. J Catal 2016, 342, 1–16. https://doi.org/10.1016/J.JCAT.2016.07.010.

(24) Paredes-Nunez, A.; Lorito, D.; Burel, L.; Motta-Meira, D.; Agostini, G.; Guilhaume, N.; Schuurman, Y.; Meunier, F. CO Hydrogenation on Cobalt-Based Catalysts: Tin Poisoning Unravels CO in Hollow Sites as a Main Surface Intermediate. Angewandte Chemie 2018, 130 (2), 556–559. https://doi.org/10.1002/ANGE.201710301.

(25) Matsubu, J. C.; Yang, V. N.; Christopher, P. Isolated Metal Active Site Concentration and Stability Control Catalytic CO_2 Reduction Selectivity. J Am Chem Soc 2015, 137 (8), 3076–3084. https://doi.org/10.1021/JA5128133.

(26) Parastaev, A.; Muravev, V.; Osta, E. H.; Kimpel, T. F.; Simons, J. F. M.; van Hoof, A. J. F.; Uslamin, E.; Zhang, L.; Struijs, J. J. C.; Burueva, D. B.; Pokochueva, E. V.; Kovtunov, K. V.; Koptyug, I. V.; Villar-Garcia, I. J.; Escudero, C.; Altantzis, T.; Liu, P.; Béché, A.; Bals, S.; Kosinov, N.; Hensen, E. J. M. Breaking Structure Sensitivity in CO_2 Hydrogenation by Tuning Metal–Oxide

Interfaces in Supported Cobalt Nanoparticles. Nat Catal 2022, 5 (11), 1051–1060. https://doi.org/10.1038/s41929-022-00874-4.

(27) Weststrate, C. J.; van de Loosdrecht, J.; Niemantsverdriet, J. W. Spectroscopic Insights into Cobalt-Catalyzed Fischer-Tropsch Synthesis: A Review of the Carbon Monoxide Interaction with Single Crystalline Surfaces of Cobalt. J Catal 2016, 342, 1–16. https://doi.org/10.1016/J.JCAT.2016.07.010.

(28) Struijs, J. J. C.; Muravev, V.; Verheijen, M. A.; Hensen, E. J. M.; Kosinov, N. Ceria-Supported Cobalt Catalyst for Low-Temperature Methanation at Low Partial Pressures of CO_2. Angewandte Chemie 2023, 135 (5), e202214864. https://doi.org/10.1002/ANGE.202214864.

(29) Parastaev, A.; Muravev, V.; Huertas Osta, E.; van Hoof, A. J. F.; Kimpel, T. F.; Kosinov, N.; Hensen, E. J. M. Boosting CO_2 Hydrogenation via Size-Dependent Metal–Support Interactions in Cobalt/Ceria-Based Catalysts. Nat Catal 2020, 3 (6), 526–533. https://doi.org/10.1038/s41929-020-0459-4.

(30) Marrocchelli, D.; Bishop, S. R.; Tuller, H. L.; Yildiz, B. Understanding Chemical Expansion in Non-Stoichiometric Oxides: Ceria and Zirconia Case Studies. Adv Funct Mater 2012, 22 (9), 1958–1965. https://doi.org/10.1002/ADFM.201102648.

(31) Coduri, M.; Scavini, M.; Allieta, M.; Brunelli, M.; Ferrero, C. Defect Structure of Y-Doped Ceria on Different Length Scales. Chemistry of Materials 2013, 25 (21), 4278–4289. https://doi.org/10.1021/CM402359D.

(32) Pakharukova, V. P.; Potemkin, D. I.; Stonkus, O. A.; Kharchenko, N. A.; Saraev, A. A.; Gorlova, A. M. Investigation of the Structure and Interface Features of $Ni/Ce_{1-X}Zr_xO_2$ Catalysts for CO and CO_2 Methanation. Journal of Physical Chemistry C 2021, 125 (37), 20538–20550. https://doi.org/10.1021/ACS.JPCC.1C05529.

(33) Oh, R.; Huang, X.; Hayward, J.; Zheng, Y.; Chen, M.; Park, G. S.; Hutchings, G.; Kim, S. K. Insights into CeO_2 Particle Size Dependent Selectivity Control for CO_2 Hydrogenation Using Co/CeO_2 Catalysts. ACS Catal 2024, 897–906. https://doi.org/10.1021/ACSCATAL.3C05139.

(34) Franken, T.; Terreni, J.; Borgschulte, A.; Heel, A. Solid Solutions in Reductive Environment – A Case Study on Improved CO_2 Hydrogenation to Methane on Cobalt Based Catalysts Derived from Ternary Mixed Metal Oxides by Modified Reducibility. J Catal 2020, 382, 385–394. https://doi.org/10.1016/J.JCAT.2019.12.045.

(35) Zhou, G.; Liu, H.; Xing, Y.; Xu, S.; Xie, H.; Xiong, K. CO_2 Hydrogenation to Methane over Mesoporous Co/SiO2 Catalysts: Effect of Structure. Journal of CO_2 Utilization 2018, 26, 221–229. https://doi.org/10.1016/J.JCOU.2018.04.023.

(36) Shin, H. H.; Lu, L.; Yang, Z.; Kiely, C. J.; McIntosh, S. Cobalt Catalysts Decorated with Platinum Atoms Supported on Barium Zirconate Provide Enhanced Activity and Selectivity for CO_2 Methanation. ACS Catal 2016, 6 (5), 2811–2818. https://doi.org/10.1021/ACSCATAL.6B00005.

(37) Díez-Ramírez, J.; Sánchez, P.; Kyriakou, V.; Zafeiratos, S.; Marnellos, G. E.; Konsolakis, M.; Dorado, F. Effect of Support Nature on the Cobalt-Catalyzed CO_2 Hydrogenation. Journal of CO_2 Utilization 2017, 21, 562–571. https://doi.org/10.1016/J.JCOU.2017.08.019.

(38) Beaumont, S. K.; Alayoglu, S.; Specht, C.; Michalak, W. D.; Pushkarev, V. V.; Guo, J.; Kruse, N.; Somorjai, G. A. Combining in Situ NEXAFS Spectroscopy and CO_2 Methanation Kinetics to Study Pt and Co Nanoparticle Catalysts Reveals Key Insights into the Role of Platinum in Promoted Cobalt Catalysis. J Am Chem Soc 2014, 136 (28), 9898–9901. https://doi.org/10.1021/JA505286J.

(39) Iablokov, V.; Beaumont, S. K.; Alayoglu, S.; Pushkarev, V. V.; Specht, C.; Gao, J.; Alivisatos, A. P.; Kruse, N.; Somorjai, G. A. Size-Controlled Model Co Nanoparticle Catalysts for CO_2

Hydrogenation: Synthesis, Characterization, and Catalytic Reactions. Nano Lett 2012, 12 (6), 3091–3096. https://doi.org/10.1021/NL300973B.

(40) Melaet, G.; Ralston, W. T.; Li, C. S.; Alayoglu, S.; An, K.; Musselwhite, N.; Kalkan, B.; Somorjai, G. A. Evidence of Highly Active Cobalt Oxide Catalyst for the Fischer-Tropsch Synthesis and CO_2 Hydrogenation. J Am Chem Soc 2014, 136 (6), 2260–2263. https://doi.org/10.1021/JA412447Q.

(41) Nguyen, T. H.; Kim, H. B.; Park, E. D. CO and CO_2 Methanation over CeO_2-Supported Cobalt Catalysts. Catalysts 2022, 12 (2), 212. https://doi.org/10.3390/CATAL12020212/S1.

(42) Dostagir, N. H. M.; Rattanawan, R.; Gao, M.; Ota, J.; Hasegawa, J. Y.; Asakura, K.; Fukouka, A.; Shrotri, A. Co Single Atoms in ZrO_2 with Inherent Oxygen Vacancies for Selective Hydrogenation of CO_2 to CO. ACS Catal 2021, 11 (15), 9450–9461. https://doi.org/10.1021/ACSCATAL.1C02041.

(43) Zhou, G.; Zhao, S.; Xie, F.; Chen, S.; Xie, H. Construction of Surface Active Centers on the Mesoporous Co/$CeO_{2-\delta}$ Catalysts for CO_2 Hydrogenation. Int J Hydrogen Energy 2023. https://doi.org/10.1016/J.IJHYDENE.2023.04.107.

(44) Have, I. C. ten; Kromwijk, J. J. G.; Monai, M.; Ferri, D.; Sterk, E. B.; Meirer, F.; Weckhuysen, B. M. Uncovering the Reaction Mechanism behind CoO as Active Phase for CO_2 Hydrogenation. Nature Communications 2022 13:1 2022, 13 (1), 1–11. https://doi.org/10.1038/s41467-022-27981-x.

(45) Xu, S.; Xie, F.; Xie, H.; Zhou, G.; Liu, X. Effect of Structure and Composition on the CO_2 Hydrogenation Properties over Bimodal Mesoporous CeCo Composite Catalyst. Chemical Engineering Journal 2019, 375, 122023. https://doi.org/10.1016/J.CEJ.2019.122023.

(46) Wang, L.; Guan, E.; Wang, Y.; Wang, L.; Gong, Z.; Cui, Y.; Meng, X.; Gates, B. C.; Xiao, F. S. Silica Accelerates the Selective Hydrogenation of CO_2 to Methanol on Cobalt Catalysts. Nature Communications 2020 11:1 2020, 11 (1), 1–9. https://doi.org/10.1038/s41467-020-14817-9.

(47) Dai, B.; Zhou, G.; Ge, S.; Xie, H.; Jiao, Z.; Zhang, G.; Xiong, K. CO_2 Reverse Water-Gas Shift Reaction on Mesoporous M-CeO_2 Catalysts. Can J Chem Eng 2017, 95 (4), 634–642. https://doi.org/10.1002/cjce.22730.

(48) Deng, K.; Lin, L.; Rui, N.; Vovchok, D.; Zhang, F.; Zhang, S.; Senanayake, S. D.; Kim, T.; Rodriguez, J. A. Studies of CO_2 Hydrogenation over Cobalt/Ceria Catalysts with in Situ Characterization: The Effect of Cobalt Loading and Metal–Support Interactions on the Catalytic Activity. Catal Sci Technol 2020, 10 (19), 6468–6482. https://doi.org/10.1039/D0CY00962H.
